# Stabilization of Endothelial Receptor Arrays by a Polarized Spectrin Cytoskeleton Facilitates Rolling and Adhesion of Leukocytes

**DOI:** 10.1016/j.celrep.2020.107798

**Published:** 2020-06-23

**Authors:** Sivakami Mylvaganam, Magdalena Riedl, Anthony Vega, Richard F. Collins, Khuloud Jaqaman, Sergio Grinstein, Spencer A. Freeman

**Affiliations:** 1 Program in Cell Biology, Peter Gilgan Centre for Research and Learning, Hospital for Sick Children, 686 Bay Street, 19-9800, Toronto, ON M5G 0A4, Canada; 2 Department of Biochemistry, University of Toronto, 1 King’s College Circle, Toronto, ON M5S 1A8, Canada; 3 Department of Biophysics, University of Texas Southwestern Medical Center, Dallas, TX 75390, USA; 4 Lyda Hill Department of Bioinformatics, University of Texas Southwestern Medical Center, Dallas, TX 75390, USA; 5 Keenan Research Centre for Biomedical Science, St. Michael’s Hospital, Toronto, ON M5B 1W8, Canada; 6 Lead Contact

## Abstract

Multivalent complexes of endothelial adhesion receptors (e.g., selectins) engage leukocytes to orchestrate their migration to inflamed tissues. Proper anchorage and sufficient density (clustering) of endothelial receptors are required for efficient leukocyte capture and rolling. We demonstrate that a polarized spectrin network dictates the stability of the endothelial cytoskeleton, which is attached to the apical membrane, at least in part, by the abundant transmembrane protein CD44. Single-particle tracking revealed that CD44 undergoes prolonged periods of immobilization as it tethers to the cytoskeleton. The CD44-spectrin “picket fence” alters the behavior of bystander molecules—notably, selectins—curtailing their mobility, inducing their apical accumulation, and favoring their clustering within caveolae. Accordingly, depletion of either spectrin or CD44 virtually eliminated leukocyte rolling and adhesion to the endothelium. Our results indicate that a unique spectrin-based apical cytoskeleton tethered to transmembrane pickets—notably, CD44—is essential for proper extravasation of leukocytes in response to inflammation.

## In Brief

Mylvaganam et al. show that an apical CD44-spectrin “picket fence” alters the behavior of endothelial selectins, curtailing their mobility, inducing their apical accumulation, and favoring their clustering within caveolae. Both components of this picket fence are required for efficient capture, rolling, and firm adhesion of leukocytes.

## Graphical Abstract

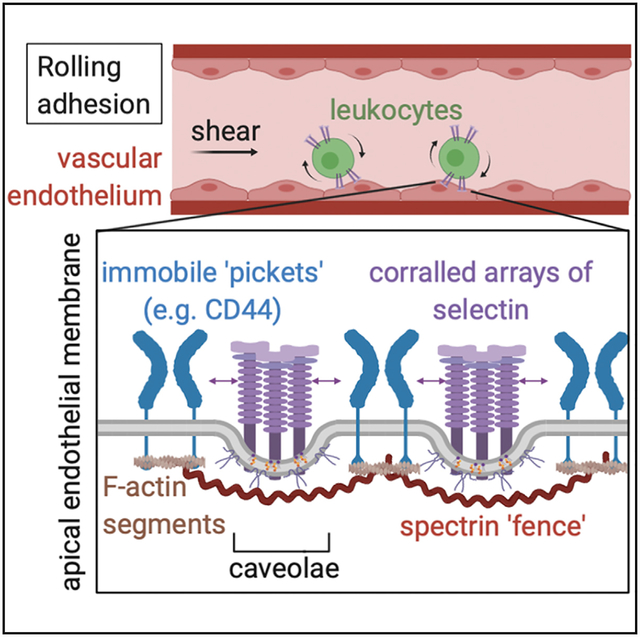

## INTRODUCTION

During inflammation, leukocytes are recruited from blood to sites of infection or injury by a highly regulated, sequential process termed “rolling adhesion.” This recruitment is initiated upon activation of endothelial cells near inflamed sites, which induces surface expression of adhesion molecules that foster interactions with circulating leukocytes. Of these, endothelial P- and E-selec-tins (CD62P/E) are of particular importance, as they are largely responsible for the initial steps of this sequence: leukocyte capture and rolling (Kunkel et al., 1997; Kunkel and Ley, 1996; Lawrence and Springer, 1991; Scalia et al., 1999). Selectins form low-affinity, high-avidity interactions with their cognate ligands (McEver, 2002; McEver et al., 1995) that can withstand high-tensile forces (Alon et al., 1997; Marshall et al., 2003) and are influenced by surface selectin density (Alon et al., 1995; Chen and Springer, 1999; Lawrence and Springer, 1991; Zhao et al., 1995). As a result, stringent regulation of the expression level, spatial organization, and stability of P- and E-selectins is paramount to ensure appropriate targeting of an immune response.

CD44 is a single-spanning transmembrane glycoprotein expressed on both leukocytes and endothelial cells and is the primary receptor for hyaluronic acid (HA), an abundant glycosaminoglycan that forms dense pericellular networks (Aruffo et al., 1990; Henry and Duling, 1999). Numerous studies report that, like selectins, CD44 is also required for proper leukocyte rolling and recruitment to inflamed tissues. CD44^−/−^ mice are protected from many inflammatory conditions (Cuff et al., 2001; Gonda et al., 2005; Lim et al., 2019; Stoop et al., 2001; Wang et al., 2002b), and defects in the rolling/adhesion cascade have been described in CD44^−/−^ animals (Bonder et al., 2006; Hidalgo et al., 2007; Khan et al., 2004; McDonald et al., 2008). Importantly, both adoptive transfer experiments and chimeric studies demonstrate a specific requirement for endothelial CD44 in rolling and adhesion (Rouschop et al., 2005; Winkler et al., 2012). While both CD44 and selectins appear to have critical functions in this pathway, the physical and functional relationships between these molecules are not understood.

The distribution and association of receptors in the plasma membrane are dictated, at least in part, by their lateral mobility. In this context, it is noteworthy that the mobility of proteins in biological membranes is relatively limited: diffusion coefficients determined in cell membranes are orders of magnitude lower than those measured in synthetic bilayers mimicking the lipid composition of cells (Kusumi et al., 2012; Kusumi and Sako, 1996). A “picket-fence” model was proposed to explain these differences, where the submembrane cytoskeletal meshwork is considered analogous to a fence tethered to defined transmembrane proteins that serve as pickets. The cytoskeletal fence and transmembrane pickets limit the diffusion of otherwise mobile proteins and can confine them into nanodomains. The identity of the transmembrane pickets is largely unknown. CD44 can form stable interactions with the cortical cytoskeleton by binding to adaptor proteins of the ezrin-radixin-moesin (ERM) or ankyrin families (Lokeshwar et al., 1994; Tsukita et al., 1994); as such, and because of its relative abundance, CD44 could, in principle, function as a picket. Indeed, CD44 was reported to regulate the diffusion and organization of phagocytic receptors in the membrane of macrophages (Freeman et al., 2018). It seemed plausible, therefore, that CD44 could similarly influence the function of selectins by limiting their mobility and/or confining them into sub-domains that may be required for effective rolling. To test these possibilities, we used single-molecule imaging in combination with genetic and pharmacological manipulations. We describe the unique properties of an apical (i.e., luminal) spec-trin-based submembranous fence that is, at least partly, tethered to CD44 in endothelial cells. This picket fence plays an important role in stabilizing functional selectin-enriched domains required for leukocyte rolling and adhesion.

## RESULTS

### CD44 Serves as a Picket in the Apical Endothelium

**Figure 1.**
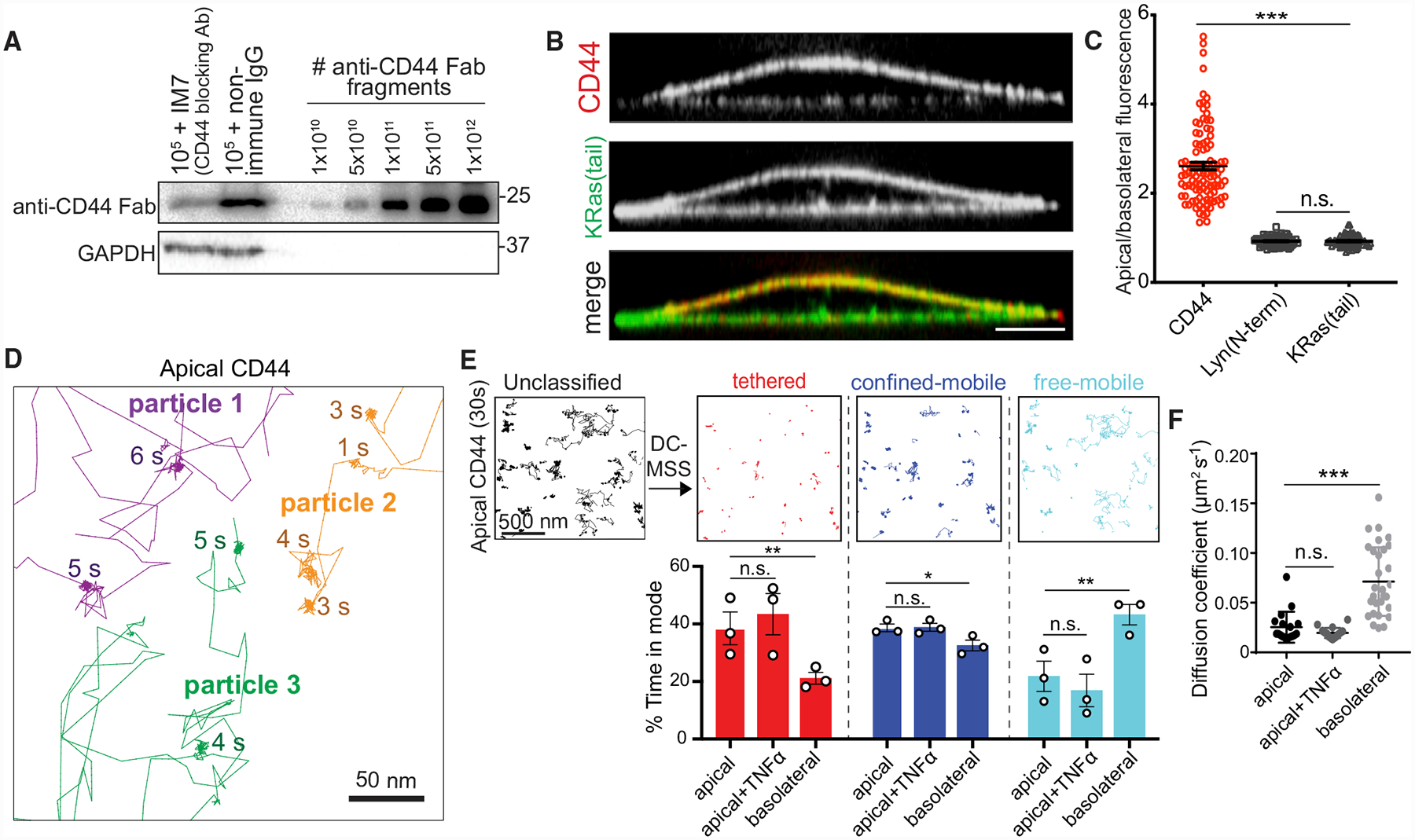
Single-Molecule Tracking Reveals Frequent Immobilization of CD44 in the Apical Endothelial Membrane (A) Immortalized HUVECs (RF24 cells) were labeled with saturating concentrations of biotinylated anti-CD44 Fab following incubation with either non-specific immuoglobulin G (IgG) or full IM7 anti-CD44 blocking antibody. Cells were subjected to electrophoresis and blotting with avidin-conjugated peroxidase and compared to known quantities of Fab. Representative of 3 experiments. (B) Orthogonal section of endothelial cell expressing KRas(tail)-GFP (green) and immunostained for endogenous CD44 (red). Here and elsewhere: scale bar, 5 μm, unless otherwise indicated. (C) Ratio of apical/basolateral membrane fluorescence of CD44 and two plasma membrane markers (KRas(tail) and the N-terminal domain of Lyn). Here and elsewhere: dots represent the mean for each cell and bars represent the overall means ± SE; n = 3; ≥30 cells per experiment. (D) Single CD44 molecules were visualized on the apical surface of RF24 cells using anti-CD44 Fab labeled with Qdots and tracked for 30 s at 33 Hz. Representative trajectories with the duration of immobilization periods are indicated. (E) Top: trajectories of Qdot-labeled CD44 particles (black) recorded for 30 s at 33 Hz (scale bar, 500 nm) were analyzed using DC-MSS and segmented as tethered (red), confined-mobile (blue), or freely mobile (cyan). Bottom: quantification of the fraction of time CD44 displays each motion type in untreated cells or in cells pre-stimulated with TNF-α for 4 h. n = 3; ≥25 cells per experiment. (F) Diffusion coefficient of CD44 determined using Cy3B-conjugated Fab fragments, recorded at 10 Hz. n = 3, ≥25 cells. Here and elsewhere: * p < 0.05; **p < 0.01; ***p < 0.001.

To have significant consequences on the mobility of neighboring membrane molecules, putative transmembrane pickets must be expressed at high density and spend a significant fraction of time immobilized, or “tethered.” Using biotin-conjugated Fab fragments of a monoclonal antibody (clone IM7; see STAR Methods) we calculated that there were ≈10^6^ molecules of CD44 on the surface of individual immortalized human umbilical vein endothelial (RF24) cells (HUVECs) (Figure 1A). Immunofluorescence revealed that the majority of these molecules were in the apical/luminal membrane of the endothelial cells (Figures 1B and 1C). A similar conclusion was reached analyzing orthogonal sections of primary human adipose microvascular endothelial cells (HAMECs), HUVECs, and blood outgrowth endothelial cells (BOECs) (Figure S1A), as well as murine vasculature (data not shown), revealing that this distribution was common to multiple endothelial cell types. In contrast, two plasma membrane markers, the N-terminal domain of Lyn and the tail of K-Ras (KRas(tail)), distributed homogeneously along the luminal and ventral/basal plasmalemma (Figures 1B and 1C). Therefore, apical accumulation of CD44 was not due to increased membrane surface area from microvillar convolution, as can occur in epithelia. The polarized distribution of CD44 was maintained following treatment with the proinflammatory cytokine tumor necrosis factor alpha (TNF-α) (Figure S1B). We concluded that the relative density of CD44 on the apical endothelial membrane—the surface that faces the vascular lumen—is high.

Next, we tracked single CD44 molecules to assess their mobility (see STAR Methods). Endothelial features labeled at sub-saturating concentrations with Cy3B-conjugated Fabs had fluorescence intensity similar to that of monodispersed Fabs on glass and underwent single photobleaching steps (Figure S1C), implying that individual Fab molecules were resolved and tracked.

Qdots, which are photostable and offer better positional accuracy than Cy3B, allowed for greater sampling frequency and detailed tracking of CD44 molecules over long acquisitions to analyze motion type. Video recordings of CD44 on the apical membrane of the endothelial cells (Video S1) showed that CD44 underwent seconds-long periods of apparent immobilization (Figures 1D and S1D) interrupted by shorter bursts of random mobility (Figure 1D).

Given the inhomogeneous nature of single CD44 trajectories, we implemented an enhanced version of moment scaling spectrum (MSS) analysis, called divide-and-conquer (DC-MSS; see STAR Methods). Using this approach, we compared the fraction of time that CD44 exists in distinct states: tethered (immobile), confined-mobile, and free-mobile (random) (Figure 1E). In the apical membrane, CD44 spent ≈40% of the time tethered and underwent free diffusion only ≈20% of the time (Figure 1E). In the basal (ventral) membrane, the reverse trend was observed (Figure 1E). Similar results were obtained using Cy3B-conjugated Fabs, yielding a significantly lower diffusion coefficient in the apical compared to the basal membrane (Figure 1F). Of note, the expression level, distribution, and diffusive behavior of CD44 were unchanged in inflamed (i.e., TNF-α-treated) endothelial monolayers (Figures 1E, 1F, and S1F). MSS analysis of apical CD44-Qdot trajectories on primary HUVEC cells yielded similar results (Figure S1E).

### Apical Retention of CD44 Is Dependent on Interactions with a Polarized Cytoskeleton

**Figure 2.**
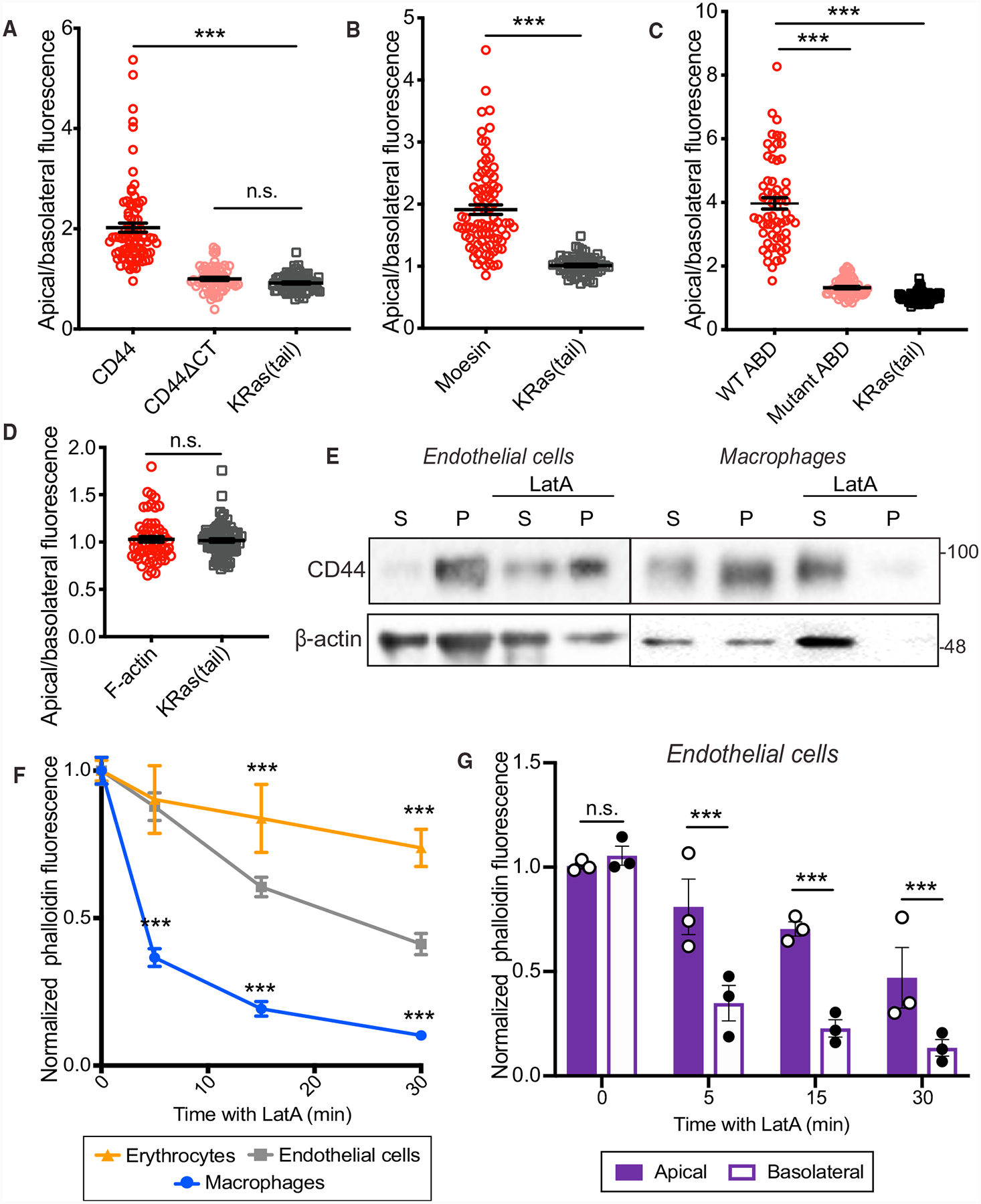
The Apical Endothelial Cytoskeleton Regulates the Distribution of Tethering Proteins (A–D) Ratio of the apical-to-basolateral membrane fluorescence of indicated proteins on untreated endothelial cells. n = 3; ≥20 cells per experiment. (A) Full-length CD44, truncated CD44 (CD44ΔCT), and KRas(tail). (B) Endogenous moesin and KRas(tail). (C) Chimeric transmembrane actin-binding domain (ABD) of ezrin, an otherwise identical construct bearing a R579A mutation in the ABD, and KRas(tail). (D) Cortical phalloidin fluorescence and KRas(tail). (E) RF24 stimulated with TNF-α for 4 h or macrophages (RAW) were untreated (left lanes) or treated with 1 μM latrunculin A (LatA) (right lanes) for 30 min, extracted in cytoskeleton-stabilizing buffer containing non-ionic detergent and centrifuged. The CD44 and actin content of the pellet (P) and supernatant (S) were analyzed by immunoblotting. n = 3. (F) Comparison of the total phalloidin fluorescence of erythrocytes, endothelial cells (RF24), or macrophages (RAW) following 5, 15 or 30 min of 1 μM LatA treatment, normalized to the fluorescence intensity of untreated cells. n = 3, ≥20 cells. (G) Comparison of phalloidin fluorescence at the apical or basolateral membrane of endothelial cells (RF24) following 5-, 15-, or 30-min treatment with 1 μM LatA, normalized to intensity at the respective membrane of untreated cells. In (F) and (G), n = 3; ≥30 cells per experiment.

Immobilization of transmembrane proteins is often associated with their ability to bind to the submembrane cytoskeleton (Tomishige et al., 1998; Trimble and Grinstein, 2015). We hypothesized that either (1) adaptors that affix CD44 to the submembrane cytoskeleton or (2) the properties of the submembrane skeletons themselves differed between the apical and basolateral cell surfaces. To test these hypotheses, we engineered mCherry-tagged wild-type (WT) CD44, as well as a form that lacks ERM- and ankyrin-binding sites due to truncation of its cytoplasmic domain (ΔCT). The tagged WT-CD44 preferentially accumulated at the apical membrane (Figures 2A and S2A). In contrast, when expressed at similar levels, CD44ΔCT distributed evenly throughout the apical and basolateral membranes (Figures 2A and S2A), suggesting that interactions between the cytosolic tail of CD44 and adaptor proteins are required for apical enrichment.

Moesin is the most abundant ERM in endothelial cells (Berryman et al., 1993), and immunofluorescence experiments revealed that it accumulates preferentially at the apical membrane (Figures 2B, S2B, and S2F). The asymmetric distribution of moesin could, therefore, account for the apical tethering of CD44. ERM proteins are targeted to membranes via their FERM domain, which binds phosphatidylinositol(4,5)-*bis*phosphate (PtdIns(4,5)P_2_) (Fehon et al., 2010). Apical accumulation of this lipid could account for the asymmetric distribution of moesin and CD44 in endothelial cells. By expressing a fluorescent PtdIns(4,5)P_2_ biosensor, the pleckstrin-homology domain of phospholipase Cδ fused to GFP (PLCδ-PH-GFP), we found that PtdIns(4,5)P_2_ was distributed homogeneously in the plasma membrane (Figure 2C), ruling out PtdIns(4,5)P_2_ polarization as the mechanism underlying the apical preference of moesin.

In addition to binding PtdIns(4,5)P_2_, ERMs also interact with transmembrane proteins and can be phosphorylated to reveal an otherwise unexposed actin-binding domain (ABD) (Fehon et al., 2010). We expressed a chimeric form of the ERM-ABD fused to a transmembrane domain that binds actin constitutively, bypassing the need for phosphorylation, to investigate the contribution of the cytoskeleton to moesin distribution. Like full-length moesin, the chimera was enriched apically (Figures 2C and S2D). This was dependent on cytoskeletal interactions, as introducing an inactivating point mutation in the ABD (R579A) resulted in a more even distribution throughout the plasma membrane (Figures 2C and S2D).

### Actin Polymers Are Stabilized in the Apical Membrane

The density of filamentous actin (F-actin) at the apical and basolateral surfaces of the cell, assessed by phalloidin staining, was not markedly different, even following activation with TNF-α (Figures 2D, S2E, and S2F). Differential accumulation of actin was therefore unlikely to account for the polarization of CD44. While the amount of F-actin is similar on both sides of the cell, its interactions with membrane proteins may differ. We assessed the stability of CD44-cytoskeleton interactions by extracting activated cells with non-ionic detergent in the presence of a cytoskeletal-stabilizing buffer (see STAR Methods). This resulted in sedimentation of a significant proportion of CD44 along with the detergent-resistant cytoskeletal fraction (Figure 2E). As expected, preventing monomeric actin incorporation into filaments with latrunculin A (LatA) increased the fraction of soluble actin. Remarkably, however, LatA did not significantly alter the fraction of CD44 extracted (Figure 2E). A significant fraction of the endothelial actin remained insoluble after treatment with LatA under these conditions (Figure 2E). These findings were in contrast to observations made in macrophages, where similar treatment solubilized virtually all actin and extracted the vast majority of CD44 (Figure 2E) (Freeman et al., 2018).

We therefore investigated differences in cytoskeletal stability by studying the resistance of F-actin to extraction following exposure to LatA for various times in endothelial cells, macrophages, and erythrocytes. In agreement with previous reports (Chasis and Mohandas, 1986; Gokhin et al., 2015), erythrocyte F-actin was largely resistant to LatA, showing only a modest loss of phalloidin intensity after 30 min (Figures 2F and S2G). In contrast, treatment with LatA for as little as 5 min caused a pronounced (≥60%) loss of phalloidin fluorescence in macrophages (Figures 2F and S2G), consistent with rapid F-actin turnover. In endothelial cells, an intermediate rate of F-actin loss was observed (Figures 2F and S2G).

Confocal analysis of the residual phalloidin fluorescence revealed a slower decrease in apical F-actin in endothelial cells with LatA, compared to basolateral F-actin (Figures 2G and S2H). We concluded that actin remodeling proceeds at very different rates on the two faces of endothelial cells. This differential stability could influence the ability of apical and basolateral cytoskeletal networks to immobilize proteins.

### Spectrin Confers Unique Features to the Apical Endothelial Membrane

**Figure 3.**
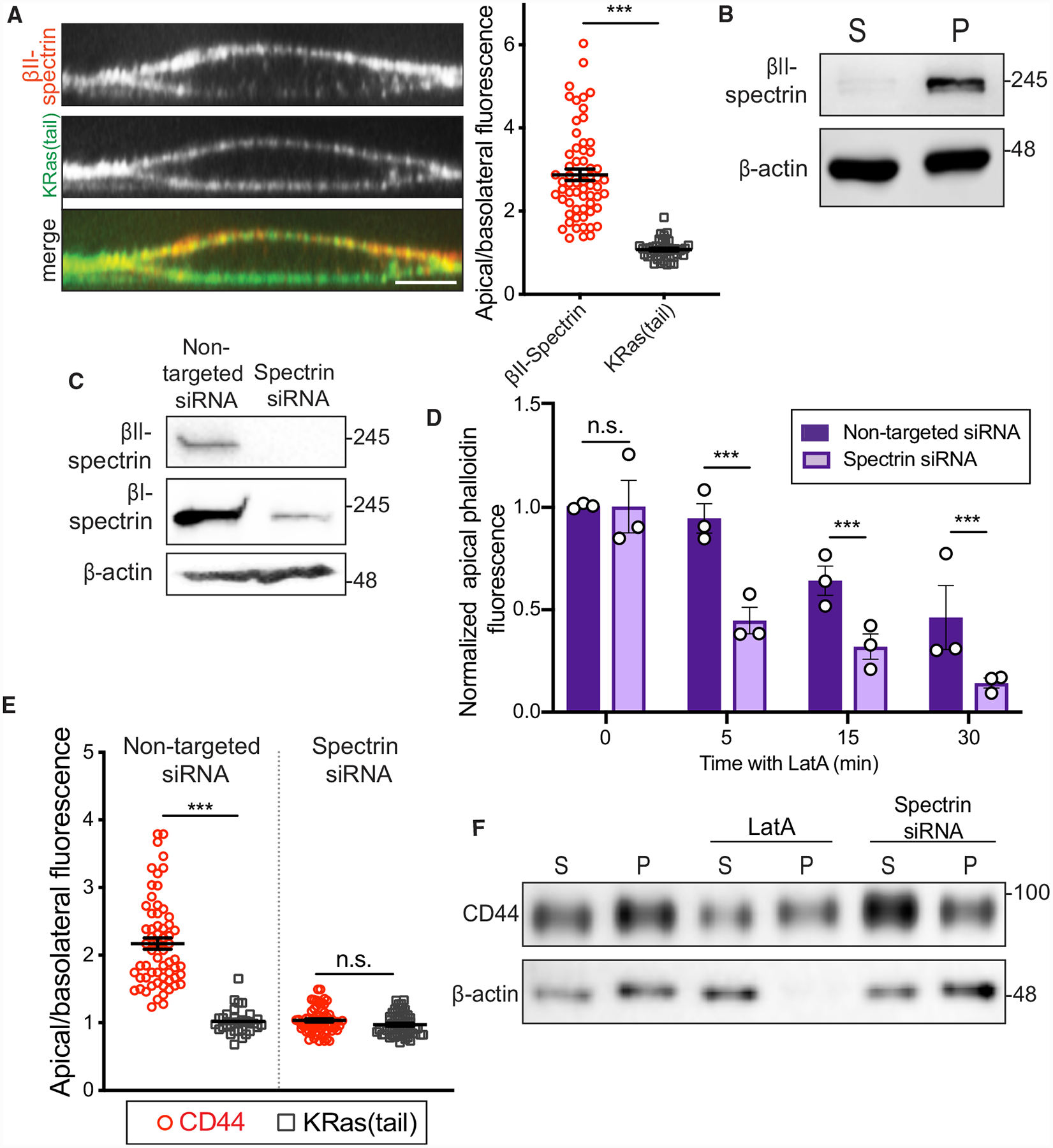
Apical Spectrins Maintain Stability of Actin Filaments and Regulate Associations with Transmembrane Proteins (A) Left: orthogonal section of representative endothelial cell expressing KRas(tail)-GFP (green) and stained for endogenous βII-spectrin (red). Right: apical/basolateral membrane fluorescence of βII-spectrin and of KRas(tail)-GFP. n = 3; ≥20 cells per experiment. (B) Activated RF24 cells were extracted as in Figure 2 and centrifuged. Pellet (P) and supernatant (S) were analyzed by immunoblotting. n = 3. (C) βI- and βII-spectrin expression in RF24 cells probed by immunoblotting after transfection with non-targeted or βI+βII-spectrin-targeted siRNAs. n = 3. (D) Comparison of phalloidin fluorescence at the apical plasma membrane of RF24 cells transfected with non-targeted or βI+βII-spectrin siRNA following 5, 15, or 30 min of 1 μM LatA treatment, normalized to apical phalloidin intensity of untreated cells. (E) Comparison of apical/basolateral fluorescence of immunostained CD44 and KRas(tail)-GFP in cells transfected with non-targeted or βI+βII-spectrin siRNAs. For (D) and (E), n = 3; ≥30 cells per experiment. (F) RF24 cells were transfected with non-targeted or βI+βII-spectrin siRNA and activated with TNF-α for 4 h. Non-targeted siRNA-transfected cells were either left untreated or incubated with 1 μM LatA for 30 min. Cells were extracted as described above and centrifuged. CD44 and actin content of pellet (P) and supernatant (S) were analyzed by immunoblotting. n = 3.

The insensitivity of the red cell cytoskeleton to LatA (Figures 2F and S2G) is attributed to spectrin networks, which comprise α- and β-subunit heterodimers that associate with short actin filaments. Because of the comparative stability of the apical endothelial cytoskeleton, we investigated the role of spectrin in these cells. The endothelial cytoskeleton contains predominantly αII/βII spectrin heterodimers (Stankewich et al., 2016; Wu et al., 2001), though there are also reports of (erythroid) βI-spectrin expression in these cells. We confirmed the expression of both types of β-spectrin in RF24 cells (Figure 3C). By immunofluorescence, we found that the βII subunit is enriched in the apical endothelial surface (Figures 3A and S3A). This subunit sedimented with the insoluble cytoskeletal fraction, demonstrating strong association within cytoskeletal polymers (Figure 3B).

Since α/β tetramers are the functional units of the spectrin cytoskeleton, depleting either subunit is an effective strategy for dismantling the entire network. Therefore, to study the role of spectrin in endothelial membrane dynamics, we silenced the expression of both β-spectrins using small interfering RNA (siRNA) (Figure 3C). Silencing β-spectrins had no significant effect on cortical F-actin abundance at the apical or basolateral membranes (Figure S3B). However, apical F-actin became more susceptible to LatA treatment following β-spectrin depletion (Figure 3D), acquiring dynamics similar to those of basolateral actin (compare Figure 3D to 2G; Figure S3C), which was unaffected by silencing spectrin, consistent with the scarcity of this protein at the base of the cells.

Given its apical localization and effects on apical F-actin stability, we speculated that spectrin might also be a key contributor to the apical tethering and distribution of CD44. While depleting β-spectrin did not alter the distribution of the plasma-membrane-associated KRas(tail), it dissipated the apical enrichment of CD44, resulting in a more symmetric distribution (Figures 3E and S3D). Additionally, compared to LatA treatment, which had minimal effects, disruption of spectrin increased the detergent-extractable fraction of CD44 from activated cells (Figure 3F).

### Spectrins Restrict Diffusion of CD44 and Stabilize the Glycocalyx

**Figure 4.**
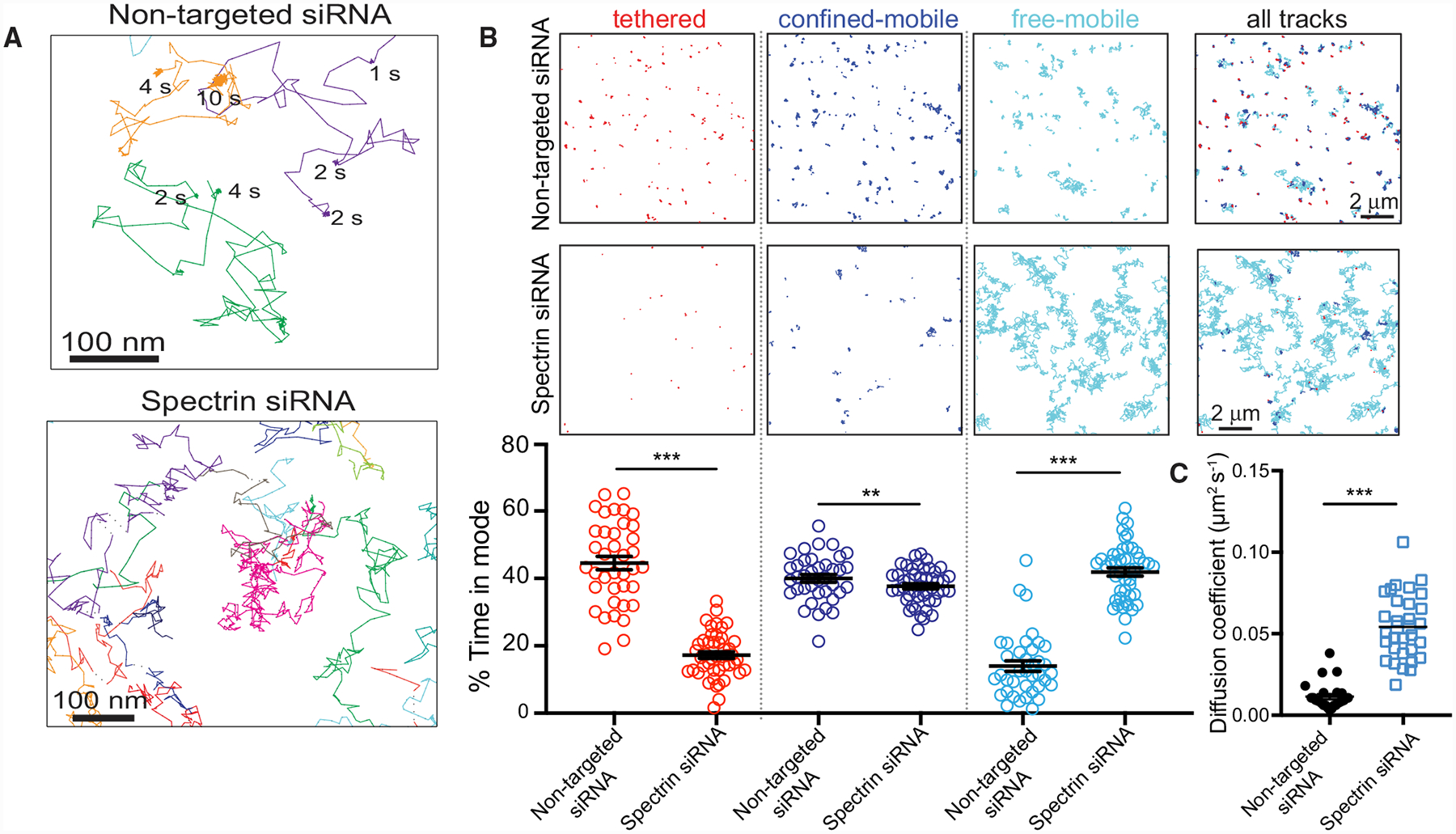
Spectrins Tether CD44 at the Apical Plasma Membrane (A) Single CD44-Qdot trajectories visualized for 30 s at 33 Hz on the apical membrane of TNF-α-activated RF24 cells transfected with non-targeted (top) or βI+βII-spectrin (bottom) siRNAs, with the duration of immobilization periods indicated. (B) Representative Qdot-labeled trajectories segmented using DC-MSS for cells transfected with non-targeted (top row) or βI+βII-spectrin siRNAs (middle row). Color coding as in Figure 1. Scale bars, 2 μm. Bottom row: fraction of time CD44 displays each motion type. n = 3; ≥20 cells per experiment. (C) Diffusion coefficient of CD44 determined using Cy3B-conjugated Fab fragments in TNF-α-activated cells transfected with control or spectrin siRNAs recorded at 10 Hz. n = 3; ≥20 cells per experiment.

We then assessed the nanoscale diffusion of CD44 by single-particle tracking following spectrin silencing. In spectrin-depleted cells, the sustained immobilization of apical CD44 observed in untreated cells was lost (Figure 4A; Video S2). DC-MSS analysis confirmed that, in spectrin-depleted cells, CD44 spent less time in the tethered and confined-mobile states while displaying increased random mobility (Figure 4B). As a result, the overall CD44 diffusion coefficient was markedly higher in spectrin-depleted cells (Figure 4C). MSS analysis on the apical surface of primary HUVECs yielded similar results (Figure S4A).

As mentioned earlier, in addition to associating with the cytoskeleton via bridging proteins, CD44 can also bind to extracellular HA (Aruffo et al., 1990), an important component of the endothelial glycocalyx (Henry and Duling, 1999). As the principal ligand for CD44, it was important to consider the potential effects of HA on CD44 tethering. Treatment with hyaluronidase removed most of the HA associated with endothelial cells (Figure S4B). However, enzymatic HA degradation had no significant effects on the diffusion or tethering frequency of apical CD44 (Figures S4C and S4D).

We also investigated whether, by tethering CD44, spectrins play a role in stabilizing the HA glycocalyx to the endothelium using biotinylated HA-binding complex (HABC), which was detected using streptavidin-conjugated Qdots. Under control conditions, the movements of HA polymers appeared restricted, displaying an average diffusion coefficient of ≈0.05 μm^2^ · s^−1^ (Figure S4E). Depletion of β-spectrins led to significantly increased movement of HA (Figure S4E).

### Apical CD44 and Spectrin Restrict Mobility of Endothelial Selectins

**Figure 5.**
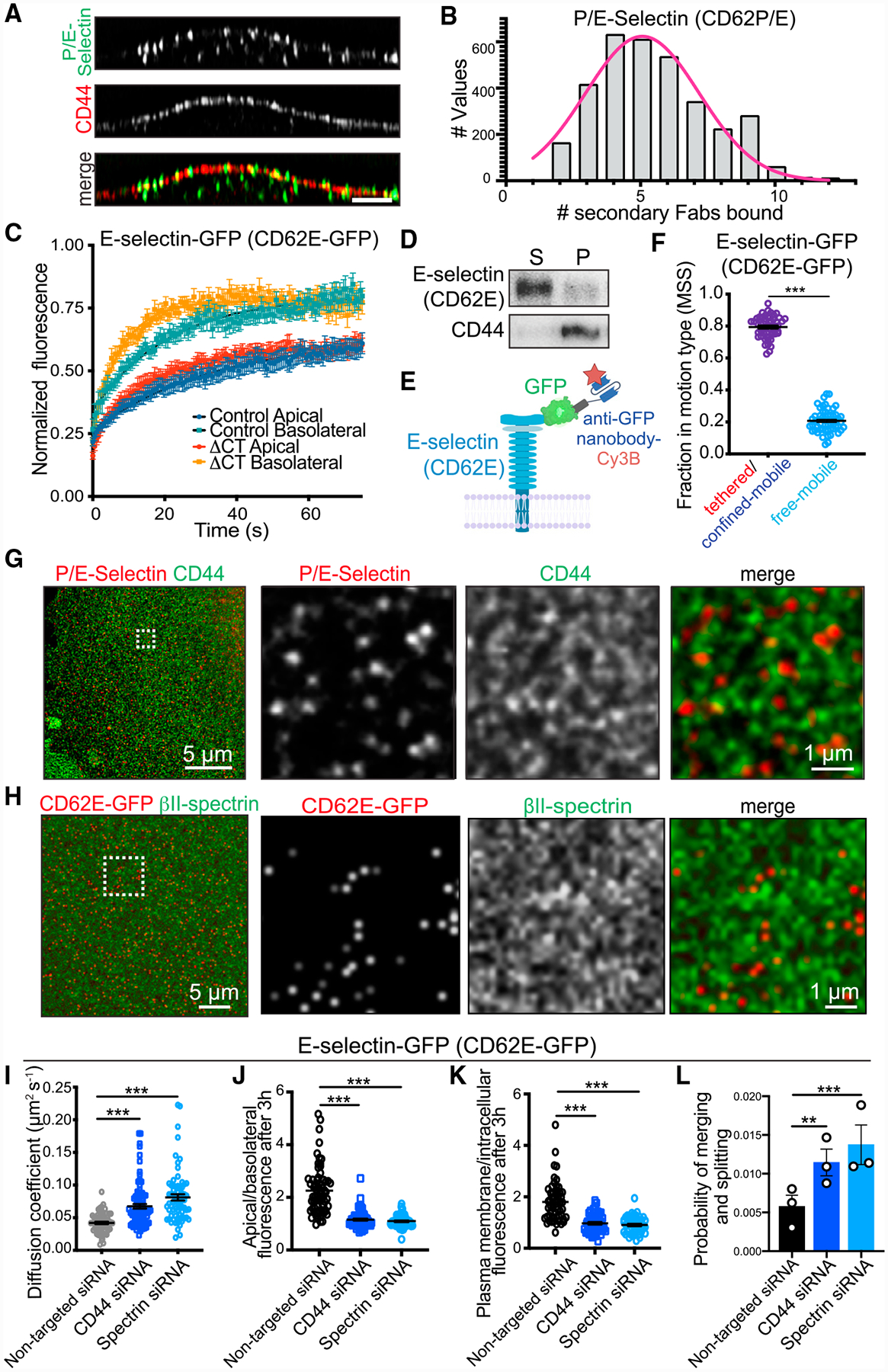
CD44 and Spectrins Restrict E-Selectin Diffusion (A) Orthogonal section of endothelial cells immunostained for CD44 (green) and P/E-selectin (CD62P/E, red) following stimulation with TNF-α for 4 h. (B) Histogram represents the estimated number of selectin molecules per cluster (see STAR Methods); n = 3; ≥15 cells per experiment. (C) Fluorescence recovery curves after photobleaching E-selectin-GFP or E-selectin-ΔCT-GFP at the apical or basolateral surface. n = 3; ≥12 cells per experiment. (D) TNF-α-activated cells extracted as in Figure 2 and centrifuged. Pellet (P) and supernatant (S) were analyzed by immunoblotting for selectin and CD44. (E) Schematic representation of fusion protein used in (F)–(K). (F) Modes of apical E-selectin-GFP mobility as determined using MSS analysis. n = 3; ≥20 cells per experiment. (G) TNF-α-activated endothelial cells stained for CD44 (green) and P/E-selectin (CD62P/E, red) and imaged by STED. (H) Endothelial cells expressing E-selectin-GFP stained with anti-GFP nanobody-DyLight-650 (red) or anti-βII-spectrin (green) and imaged by STED. (I) Mean diffusion coefficient of E-selectin-GFP in cells transfected with non-targeted, CD44-targeted, or β-spectrin-targeted siRNAs. n = 3; ≥20 cells per experiment. (J and K) Cells expressing E-selectin-GFP were labeled with anti-GFP nanobody and incubated for 3 h and visualized by confocal microscopy. Comparison of apical/basolateral fluorescence (J) or plasma membrane/intracellular fluorescence (K) in cells transfected with non-targeted, CD44-targeted, or spectrin-targeted siRNAs; n = 3; ≥20 cells per experiment. (L) The probability of a single E-selectin-GFP merging or splitting from a pre-existing cluster in 5 s. Representation of the mean probability for all E-selectin-GFP molecules in each video, comparing cells transfected with control, CD44-targeted, or β-spectrin-targeted siRNAs; n = 3; ≥20 cells per experiment.

We proceeded to study whether the spectrin-based skeleton regulates leukocyte rolling and adhesion. Consistent with earlier reports, P/E selectins formed punctate arrays on the apical surface of TNF-α-stimulated endothelial cells (Figure 5A). The punctate appearance suggested that selectins cluster on the apical surface, and we approximated that clusters consist of an average of ≈5 selectins (range = 2–10; Figure 5B).

Both P and E isoforms of selectin are expressed on the surface of activated endothelial cells, and their functions are largely redundant (McEver, 2002). We focused primarily on the effects of CD44 and spectrin on the mobility and distribution of E-selectin, due to its more robust and sustained expression pattern (Kunkel et al., 1997; McEver, 2002). Using fluorescence recovery after photobleaching (FRAP), we found that the mobility of E-selectin was limited on the apical, but not basolateral, surface of endothelial cells (Figures 5C and S5A). FRAP measurements that determine the behavior of an ensemble of selectins were complemented by single-molecule tracking. Because we were unable to obtain Fab fragments of CD62P/E antibodies with sufficiently high affinity, we developed a system whereby we could study the dynamics of single CD62E conjugated to exofacial GFP (see STAR Methods; Figures 5E and S5B). Using this system, analysis of apical trajectories found that approximately 80% of selectin molecules exhibited immobile/confined dynamics (as determined using MSS; Figure 5F) in concordance with the FRAP data.

Direct association with CD44 and/or the spectrin cytoskeleton could, in principle, account for restricted selectin mobility. However, we found that truncation of its cytoplasmic domain did not significantly alter E-selectin mobility (Figure 5C). Moreover, CD62E associated only marginally with cytoskeletal fractions following extraction (Figure 5D).

When analyzed by diffraction-limited confocal microscopy, the apical clusters of selectins did not colocalize significantly with CD44 (Figure 5A), which distributed evenly across an endothelial monolayer (Figure S5C). More detailed observations using stimulated emission depletion (STED) microscopy revealed that CD44 and βII-spectrin each formed a dense apical meshwork that appeared to enclose but not colocalize with selectin puncta (Figures 5G and 5H). In fact, the Pearson’s coefficients from multiple determinations of βII-spectrin and selectin distribution were statistically different from those obtained for comparable numbers of randomly distributed features, implying that the mutual exclusion of the two proteins is significant (Figures S5D and S5E).

This supports a role for CD44 and spectrin in corralling selectins. In accordance with this hypothesis, E-selectin-GFP diffusion was significantly higher in endothelial cells lacking CD44 or β-spectrins (Figure 5I).

### Restricting Selectin Mobility Is Required to Maintain Its Apical Density and Clustering

To test how spectrin and CD44 regulate the apical enrichment of selectins, we compared the behavior of selectin (CD62E-GFP) in control, CD44-depleted, or spectrin-depleted cells. The mobility and fate of the selectin were monitored following addition of fluorescent anti-GFP nanobody to live cells. Immediately after addition, the nanobody was predominantly localized on the apical membrane, to which it had unimpeded access. However, in cells lacking CD44 or spectrin, a large fraction of the labeling nanobody distributed to the basolateral membrane or was internalized after 3 h (Figures 5J, 5K, and S5F). As a result, the ratio of apical to basolateral fluorescence (i.e., selectin) was markedly lower—approaching unity—in CD44- or spectrin-depleted cells (Figure 5J), as was the ratio of plasmalemmal to intracellular fluorescence (Figure 5K).

The stability of selectin clusters was then assessed by analyzing single-molecule trajectories using a merge-split detection algorithm. Despite the lower density of apical selectins following CD44 or spectrin knockdown, the frequency of merging-splitting events was greater in the absence of CD44 or spectrin (Figure 5L), implying lower stability of clusters in these conditions.

### Apical CD44 and Spectrin Restrict the Mobility of Endothelial Caveolae

**Figure 6.**
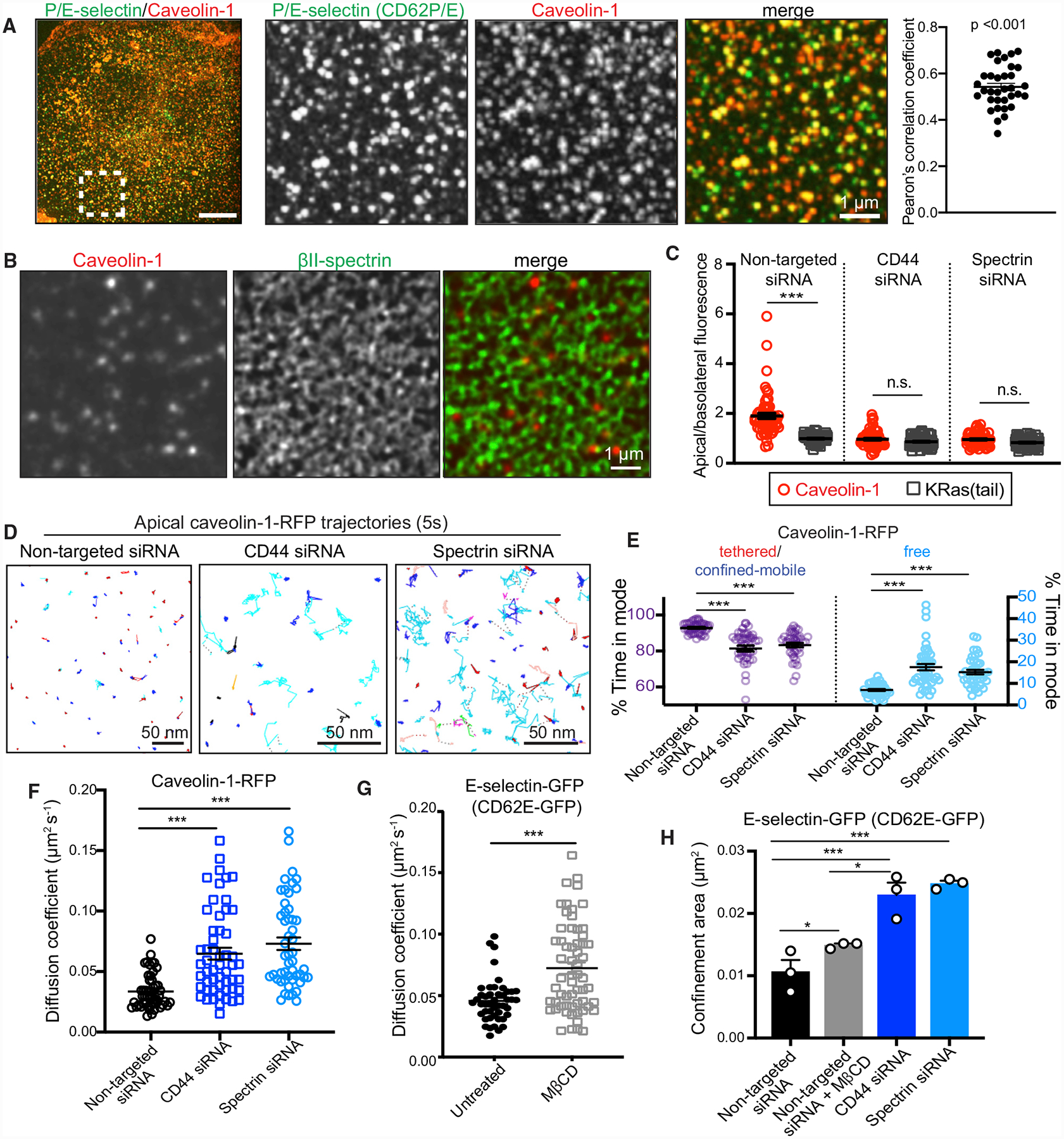
CD44 and Spectrin Restrict Caveolar Mobility which, in Turn, Regulates Diffusion of Selectin (A) TNF-α-activated endothelial cells immunostained for P/E-selectin (green) and caveolin-1 (red). Area denoted in left panel is magnified in center panels (scale bars, 1 μm). Pearson’s correlation coefficient of individual cells is indicated in the far-right panel. (B) Distribution of caveolin-1 (red) and βI+βII-spectrin (green) imaged using STED. Scale bar, 1 μm. (C) Ratio of apical/basolateral fluorescence of immunostained caveolin-1 and KRas(tail)-GFP in cells treated with non-targeted, CD44, or βI+βII-spectrin siRNA. n = 3; ≥25 cells per experiment. (D) Representative trajectories of apical caveolin-1-RFP under the indicated conditions. Color coding is as in Figure 1. Scale bars, 50 nm. (E) DC-MSS classification of caveolin-1-RFP trajectories as in (D) for cells transfected with non-targeted, CD44, or βI+βII-spectrin siRNAs. n = 3; ≥25 cells per experiment. (F) Diffusion coefficient determined for apical caveolin-1-RFP of non-targeted siRNA, CD44-transfected, or βI+βII-spectrin siRNA-transfected cells; n = 3, ≥25 cells. (G) Diffusion coefficients for E-selectin-GFP tagged with anti-GFP-Cy3B nanobodies in cells treated with vehicle or 10 mM methyl-β-cyclodextrin (MβCD) for 30 min. n = 3; ≥20 cells per experiment. (H) Confinement areas for E-selectin-GFP in cells treated with non-targeted, CD44, or βI+βII-spectrin siRNA ± 10 mM MβCD; n = 3; ≥20 cells per experiment.

Lipid rafts have been implicated in regulating selectin organization and leukocyte adhesion to the endothelium (Abbal et al., 2006; Setiadi and McEver, 2008), and immunofluorescence of TNF-α-activated cells revealed significant colocalization of CD62P/E and caveolin-1, a primary constituent of caveolae (Figure 6A; Pearson’s colocalization coefficient, ≈0.6, p < 0.001). We reasoned that CD44 and spectrin could exert their effects on selectins by altering caveolar behavior. Using STED microscopy, we noted that, like the selectins, caveolae were corralled within CD44 and spectrin networks (Figures 6B and S6A). While present on both the apical and basolateral membranes, caveolae were significantly enriched in the apical surface, as described previously (Drab et al., 2001; Oh et al., 2007) (Figures 6C and S6B). This polarized distribution was lost upon depletion of CD44 or spectrin networks (Figures 6C and S6B).

We next examined caveolar mobility by tracking caveolin-1-RFP at the apical surface. In agreement with previous reports (Hirama et al., 2017), we found that, in untreated cells, the majority of apical caveolae were immobile most of the time (Figures 6D and 6E). In the absence of CD44 or spectrin, there was a significant increase in the fraction of caveolae undergoing random diffusion (Figures 6D and 6E) and a corresponding increase in their diffusion coefficient (Figure 6F). Therefore, changes of caveolar mobility could potentially explain the altered mobility of E-selectin.

### Caveolar Dynamics Regulate E-Selectin Diffusion

Caveolae can be disrupted by the extraction of membrane cholesterol using methyl-β-cyclodextrin (MβCD). Treatment of endothelial cells with MβCD increased the mobility of E-selectin-GFP, confirming that caveolae/rafts play a role in selectin organization (Figure 6G). MβCD also increased the average confinement area of E-selectin-GFP (Figure 6H). However, this increase was relatively modest, likely because the selectins remained confined within the picket fence. Supporting this notion, we found that disrupting the CD44/spectrin network resulted in a much greater increase in the size of individual confinement areas (Figure 6H). Moreover, when estimated using STED microscopy, the size of the individual corral areas delimited by CD44 and spectrin averaged 0.012 μm^2^, both under steady-state and inflammatory conditions (Figures S6D–S6F), similar to the confinement area of the selectins following cholesterol extraction.

### Endothelial CD44 and Spectrin Are Required for Leukocyte Rolling and Adhesion

**Figure 7.**
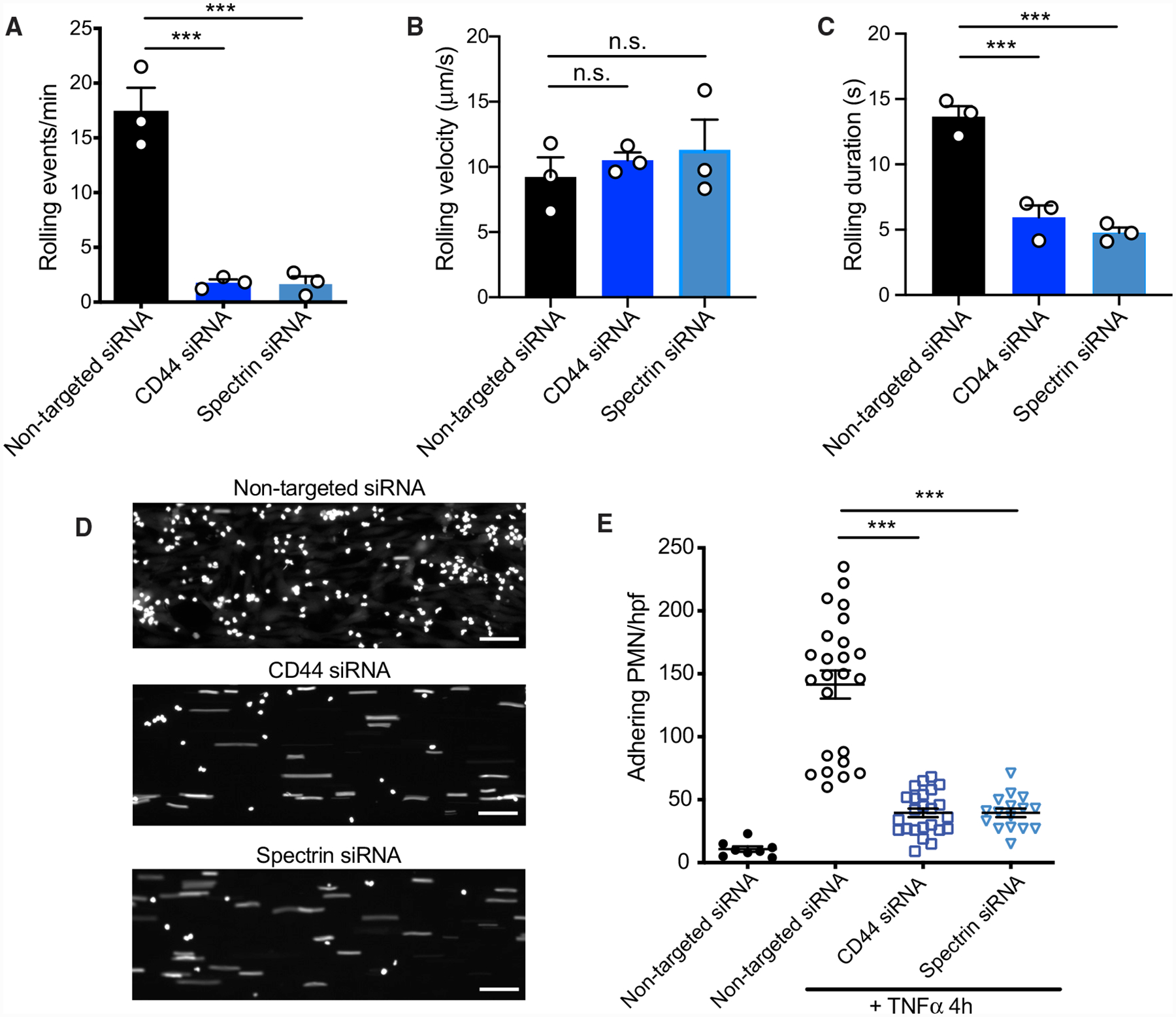
Endothelial CD44 and Spectrin Orchestrate Neutrophil Rolling and Firm Adhesion Endothelial cells treated with non-targeted, CD44, or βI+βII-spectrin siRNAs were grown in collagen-coated microfluidic channels and incubated with TNF-α for 4 h. Rolling of PMNs was determined under a constant shear rate of 1 dyne/cm^2^, as described in STAR Methods. n = 3. (A–C) Analysis of number of rolling events (A), rolling velocity (B), and rolling duration (C) from 5-min videos; n = 3. (D) PMN firm adhesion on activated endothelial cells expressing non-targeted, CD44, or βI+βII-spectrin siRNAs after 5 min of flow. Scale bars, 50 μm. (E) Firm-adhesion events per high-power field (hpf) after 5-min perfusion, as described in STAR Methods. n = 3, ≥ 5 fields.

While a general requirement for CD44 in leukocyte extravasation has been established, the underlying mechanism and possible role of its association with spectrin have not been explored. To address this, we studied the interactions between polymorphonuclear leukocytes (PMNs) and endothelial cells under hydrodynamic conditions (shear rate of 1 dyne/cm^2^) mimicking those in the microvasculature (Barabino et al., 1997; Zhao et al., 2001), with a microfluidic BioFlux system. Our experiments confirmed that depletion of CD44 was sufficient to abrogate PMN rolling almost completely (Figure 7A). Depletion of endothelial spectrin was also sufficient to drastically reduce the number of rolling events to comparable levels (Figure 7A). This reduction in rolling frequency was not accompanied by a change in rolling velocity in either condition (Figure 7B). Instead, we observed that the average duration of individual rolling events decreased in the absence of CD44 or spectrin (Figure 7C). The number of PMNs that adhered firmly onto CD44- or spectrin-depleted endothelium also decreased, which was likely secondary to rolling defects (Figures 7D and 7E). Taken together, these observations suggest that, in the absence of spectrins, freely mobile CD44 that is rarely tethered (Figure 4) is insufficient to support normal leukocyte rolling and adhesion.

## DISCUSSION

CD44 was identified as having a role in leukocyte extravasation decades ago from neutralizing antibody studies (DeGrendele et al., 1996, 1997) that were later confirmed in genetic deletion models (Bonder et al., 2006; Gonda et al., 2005; Hidalgo et al., 2007; Khan et al., 2004; McDonald et al., 2008). A few contradictory studies reported increased leukocyte infiltration in the absence of CD44 (Teder et al., 2002; Wang et al., 2002a); these are conceivably attributable to endothelial injury. Some inflammatory insults compromise vascular integrity, an effect that may be aggravated in CD44-deficient mice where impaired phagocytic clearance of immunostimulatory debris or low-molecular-weight HA could result in more prolonged and intense inflammation (Teder et al., 2002). Without an intact vascular barrier, circulating leukocytes can circumvent the tightly regulated rolling adhesion cascade and exit vessels along with the bulk flow of other blood components.

Thus while CD44 has generally been recognized as critical for regulated leukocyte extravasation, its mode of action remained undefined. In one model, endothelial CD44 was proposed to maintain a stable layer of HA onto which leukocyte CD44 can attach, mediating rolling (Khan et al., 2004; Puré and Cuff, 2001). However, this model fails to account for why the endothelium must be activated for rolling to occur. In fact, inflammatory stimuli also induce shedding of the endothelial glycocalyx (Chappell et al., 2009; Mulivor and Lipowsky, 2004), likely reducing the availability of luminal HA.

Here, we propose an alternative model to account for the role of endothelial CD44. Our findings suggest that, by tethering to an apical spectrin-based cytoskeleton, CD44 regulates rolling/adhesion by restricting the mobility of the selectins. Concordant with this model, we found that: (1) CD44 is largely immobile, tethered to the cytoskeleton in a spectrin-dependent manner (Figure 4); (2) silencing either CD44 or spectrin enhanced the mobility apical selectins (Figure 5); and (3) dismantling the apical spectrin skeleton prevented leukocyte rolling and adhesion (Figure 7). Interestingly, modification of ankyrin or ERM binding sites on the cytoplasmic domain of CD44 of leukocytes was also found to impair rolling (Wang et al., 2014). Therefore, appropriate tethering of CD44 appears essential for both cell types involved in regulated extravasation.

By confining the selectins within corrals, the spectrin-based endothelial picket fence curtails their diffusion, limiting their endocytosis and redistribution to the basolateral membrane. This has two important consequences: it increases the retention of selectins on the apical membrane and fosters the formation of high-avidity multimolecular clusters that can better secure leukocytes to the surface of the endothelium. The ability of selectins to form clusters may be further promoted by their tendency to partition into cholesterol-rich rafts or caveolae, which are themselves enriched and confined apically by spectrins (Figure 6). By these mechanisms, restricted apical mobility can increase the probability of opportune collisions between selectins and their ligands in a manner that mediates initial leukocyte capture but can also ensure that new leukocyte-endothelial contacts are continuously formed, as is observed during leukocyte rolling (Figures 7A and 7C). The transit time of rolling leukocytes has been identified as a key determinant of the efficiency of leukocyte recruitment *in vivo* (Jung et al., 1998), as it probably dictates the likelihood of inside-out activation of leukocyte integrins required for firm adhesion (Ley et al., 2007). Our findings are, therefore, relevant to *in vivo* systems where moderate increases in the frequency of rolling events preceded defects in adhesion and extravasation (Hutás et al., 2008; Szántó et al., 2004). In certain low-shear environments, CD44 deletion may still allow transient leukocyte-endothelial interactions, but other defects in rolling (e.g., its duration) may exist, explaining the observed decreases in adhesion and extravasation in these settings.

Selectin-ligand bonds that mediate slow rolling exhibit unique catch-slip dynamics characterized by remarkably high-tensile strength and duration, which are required to resist the shear forces encountered in the vascular environment (Chen and Springer, 1999; Marshall et al., 2003; Puri et al., 1997). This behavior can be attributed, at least partly, to the multivalency of the interactions (Chen and Springer, 1999), which are ostensibly facilitated by the selectin clustering described earlier. Others have postulated that anchorage to the cytoskeleton is required for selectins to bear strong tensile forces (Alon et al., 1995). Our experiments did not support the existence of direct, stable associations between selectins and the cytoskeleton, at least when unengaged by cognate ligands. Perhaps, by restricting the lateral movement of selectins, the firmly tethered spectrin meshwork functions like the proposed cytoskeletal anchor: by trapping and immobilizing the engaged receptors or the caveolae where they reside, the cytoskeleton enables the transmission of the shear force exerted on the leukocytes to the endothelial selectins, which, as a result, may alter their structure and affinity toward their ligand, buttressing the attachment and resulting in rolling.

While CD44 clearly plays an important role in regulating receptor mobility in endothelial cells, other transmembrane proteins are also likely to function as pickets and regulate selectin functions. ICAM and VCAM are especially interesting to consider. Expression of these proteins is also induced by inflammatory stimuli on the endothelial cell surface, but, unlike selectins, they can interact directly with ERM proteins and, by extension, the cytoskeleton (Barreiro et al., 2002). This may endow ICAM and VCAM with additional roles in the traffic of leukocytes beyond directly mediating their adhesion.

The preferential accumulation of CD44 and of selectins on the apical membrane is associated with, and seemingly due to, the highly polarized distribution of spectrin in multiple endothelial cell types (Figures 3A and S3A). From our experiments, it appears that spectrins serve to stabilize the actin filaments that bind these proteins (Figures 2F, 2G, and 3D). It is also plausible that certain F-actin nucleators preferentially make these spec-trin-associated filaments. It will be important to identify the determinants of apico-basolateral distribution of endothelial spectrin and to improve our understanding of how spectrin-actin networks are assembled in different cells. In other asymmetric tissue barriers such those formed by epithelial cells, apico-basolateral polarity is attributed to spatial cues established by junctional complexes (Rodriguez-Boulan and Macara, 2014) and/or by the uneven distribution of phosphoinositides (Freeman et al., 2018; Thapa and Anderson, 2012). However, the density of PtdIns(4,5)P_2_ seemed comparable in the apical and basolateral membranes of the endothelial cells used in our studies (Figure S2C). Moreover, we have observed predominantly apical distribution of CD44, moesin, caveolin-1, and spectrin, even in endothelial cells that did not form junctions with neighboring cells (data not shown). Junction-independent determinants of polarity may be particularly important in the case of endothelia, where inflammatory stimuli are associated with increased vascular permeability and disruption of endothelial junctions (Pober and Sessa, 2007). Maintaining asymmetry of the receptors that guide the transmigration of leukocytes would seem essential to facilitate their dissociation after they have traversed the vascular barrier.

In summary, we have identified the endothelial spectrin cytoskeleton as a novel regulator of immune cell diapedesis. Our findings also provide additional evidence for the role of CD44 as a transmembrane picket and demonstrate that it controls the mobility and aggregation of adhesion receptors like selectins, thereby regulating rolling and adhesion of leukocytes.

## STAR★METHODS

### RESOURCE AVAILABILITY

#### Lead Contact

Further information and requests for resources and reagents should be directed to and will be fulfilled by the Lead Contact, Spencer Freeman (spencer.freeman@sickkids.ca).

#### Materials Availability

All unique/stable reagents generated in this study are available from the Lead Contact.

#### Data and Code Availability

This study did not generate any unique datasets or code.

### EXPERIMENTAL MODEL AND SUBJECT DETAILS

Human immortalized umbilical vein endothelial cells (RF24 (Fontijn et al., 1995)), primary human umbilical vein endothelial cells (HUVEC) and primary human adipose microvascular endothelial cells (HAMEC) were grown in endothelial growth medium (EGM-2) containing 5% heat-inactivated fetal calf serum (FCS) with supplements (endothelial cell growth supplement, epidermal growth factor, basic fibroblast growth factor, heparin, hydrocortisone; PromoCell). Blood outgrowth endothelial cells (BOEC) were isolated as previously described (Noone et al., 2016) and grown in EGM-2. RAW 264.7 cells were cultured in DMEM with L-glutamine containing 10% heat-inactivated FCS as described (Freeman et al., 2018). Cell cultures were maintained at 37°C in a 5% CO_2_ incubator with 95% humidity.

### METHOD DETAILS

#### Single-particle labeling and tracking of CD44, caveolin-1 and E-selectin-GFP

Anti-CD44 monovalent Fab fragments were generated with the ImmunoPure Fab Preparation Kit (Pierce), using 2 mg of the monoclonal antibody from the IM7 hybridoma cell line as described (Freeman et al., 2018). Fab fragments were directly labeled with the succinimidyl esters of Cy3B or biotin. To label single CD44 molecules, cells were incubated with 10 ng/mL of Cy3B- or biotin-conjugated anti-CD44 Fab fragments for 10 min at 4°C. Cells labeled with biotinylated Fabs were washed in cold PBS containing Ca^2+^ and Mg^2+^, then incubated with streptavidin-655 Qdots (1:10,000 dilution) for 5 min at 4°C, to minimize lateral mobility and clustering during labeling. Cells were subsequently washed with EGM-2 containing excess biotin to block unoccupied avidin sites and prevent cross-linking. Cells labeled with Cy3B-conjugated Fab fragments were washed in PBS at 25°C. Single cells were then warmed to 37°C and imaged immediately.

To track selectin movements, anti-GFP nanobodies (V_H_H fragments) were directly labeled with the succinimidyl ester of Cy3B. Cells were transfected to express E-selectin conjugated to an exofacial GFP and incubated with 25 ng/mL of Cy3B-conjugated nanobodies for 10 min. Cells labeled with Cy3B-conjugated nanobodies were washed in PBS at 25°C. Single cells were then warmed to 37°C and imaged immediately. The nanobody specifically labeled transfected cells (Figure S5B).

To track caveolae movements, cells were transiently transfected with plasmids encoding caveolin-1-RFP.

Particle trajectories were imaged on a Zeiss Axiovert 200M microscope, with a 100x NA 1.45 oil objective, a custom 2.4x magnification lens, and a back-thinned EM-CCD camera (C9100-13, Hamamatsu). For Qdot-labeled particles, acquisitions were performed at 33 Hz using Volocity software (Perkin-Elmer). For Cy3B-labeled particles, acquisitions were performed at 10 Hz. Single particles labeled with Cy3B were detected and tracked as described (Jaqaman et al., 2008).

#### Single-particle tracking analysis

Unless otherwise indicated, diffusion coefficients were determined using a moment scaling spectrum (MSS) analysis and motion types were determined using the divide-and-conquer moment scaling spectrum (DC-MSS) analysis as described (Freeman et al., 2018; Jaqaman et al., 2011; Vega et al., 2018). Briefly, DC-MSS: 1) segments trajectories in order to classify the diffusion model of the individual segments (i.e., transients) and 2) distinguishes immobility from confined diffusion (Vega et al., 2018). To confirm correct ‘tethering’ classification, confinement area of ‘tethered’ particles was compared with the positional accuracy of Q-dots and comparable to the “movement” observed in chemically-fixed cells (Figure S1D). Diffusion coefficients and confinement areas were determined using MSS analysis. A two-step particle tracking algorithm was employed to detect merging and splitting events (Jaqaman et al., 2011).

#### Immunofluorescence

Endothelial cell monolayers were grown on coverslips coated with 50 μg/mL collagen. Cells were fixed with 4% PFA (Electron Microscopy Sciences), permeabilized with 0.1% Triton X-100 in PBS, blocked with 2% BSA in PBS, and stained with the indicated antibodies or fluorescent phalloidin in blocking solution (see Reagents and Plasmids below). After staining, coverslips were mounted onto glass slides using ProLong Diamond (Life Technologies) or imaged directly in PBS.

#### Transfection

Transfection of RF24 cells using the Neon electroporation system (Invitrogen) was performed according to the manufacturer’s recommended protocol.

#### Reagents and plasmids

Where specified, endothelial cells were incubated for 4 h in 10 ng/mL of TNFα in complete medium. Latrunculin A (Sigma) was used at 1 μM for 5–30 min for cytoskeletal fractionation and cytoskeletal stability experiments. Antibodies for immunostaining were used as follows: rat anti-human CD44 (Abcam, clone IM7) at 1 μg/mL, mouse anti-human βII-spectrin (Santa Cruz Biotechnology) at 1 μg/mL, mouse anti-human P/E-selectin (R&D systems) at 5 μg/mL, rabbit anti-human moesin (Abcam) at 1 μg/mL, rabbit anti-human caveolin-1 (BD Biosciences) at 1 μg/mL. Cy3-, Alexa 488- or Alexa 647-conjugated donkey anti-mouse or anti-human secondary antibodies (Jackson ImmunoResearch) were used at 1 μg/mL. Rhodamine- or Alexa 488-phalloidin (Life Technologies) were used at 1:1000. Coverslips were coated with rat-tail collagen type I (Sigma) at 50 μg/mL in PBS at 25°C for 1 h. Isolation and purification of the hyaluronan-binding complex (HABC) from bovine articular cartilage, and its biotinylation were described earlier (Tammi et al., 1994). Labeling of HABC with Alexa 647 was as in Rilla et al. (2008). Fluorescent HABC was added to live cells at 10 μg/ml of medium and imaged after 5 min. Hyaluronidase from bovine testes (Sigma) was used at 20 units/mL for 10–30 min at 37°C in DMEM.

CD44wt, CD44ΔCT, PLCδ(PH)-GFP, and the transmembrane I actin-binding domain of ezrin with an N-terminal hemagglutinin tag were used as previously described (Freeman et al., 2018). Caveolin-1-RFP was previously described (Hirama et al., 2017). Stealth RNAi™ siRNAs (ThermoFisher) were electroporated at 100 pM using the Neon transfection system.

#### Stimulated emission depletion (STED) microscopy

STED was performed using a Leica TCS SP8 STED 3X microscope. Images were acquired using HyD detectors and a 90X/1.4 glycerol objective. Samples were labeled with Alexa 488- or Cy3-conjugated secondary antibodies or DyLight 650 anti-GFP nanobody and excited at 499, 554 and 652 nm respectively using a white light laser. Emissions were time-gated 0.5–6 ns. Three 1.5 W depletion lasers at 592, 660 and 775 nm were used for the green, red and far-red channels, respectively, at 30%–60% of maximal power. Images were acquired using Leica LAS X software with a *xy* pixel size of 31 nm and *z* step of 200 nm. Acquired images were deconvolved with Leica Lightning software (Scientific Volume Imaging, the Netherlands).

#### Quantification of surface CD44

Confluent RF24 cells were washed with ice-cold PBS, subsequently incubated with saturating concentrations (20 μg/mL) of biotinylated anti-CD44 Fab fragments for 10 min on ice to prevent endocytosis, and then washed three times with cold PBS before extraction with lysis buffer (150 mM sodium chloride, 1% NP-40, 0.5% sodium deoxycholate, 0.1% SDS, 50 mM Tris, pH 8.0). Sample buffer was added to lysates or known quantities of Fab fragments before separation by PAGE under reducing conditions. Immunoblotting was performed with streptavidin-HRP (Cell Signaling Technology) or mouse anti-GAPDH (Abcam).

#### Quantification of selectin clusters

RF24 cells were activated for 4 h with TNFα to induce surface selectin expression, then fixed with 4% PFA, incubated with anti-P/E-selectin monoclonal antibody and subsequently labeled with a Cy3-conjugated secondary Fab fragment and imaged by confocal microscopy. Assuming that each emitting Fab was indicative of a single selectin molecule, fluorescence of surface cellular puncta was compared to fluorescence of monodispersed secondary Fab on glass using Volocity analysis software.

#### Cytoskeletal fractionation

Cells were incubated for 1 min with cytoskeletal-stabilizing buffer (4 M glycerol, 25 mM Pipes pH 6.9, 1 mM ATP, 1 mM EGTA, 1 mM MgCl_2_) and 0.1% Triton-X followed by centrifugation for 3 min at 13,000 g at 4°C. The volume of the supernatant (S), representing the Triton-soluble fraction was measured and the pellet (P), representing the Triton-insoluble cytoskeletal fraction, was resuspended in the same volume of the cytoskeletal stabilizing buffer. S and P were separated under nonreducing conditions and analyzed by immunoblotting. Antibody binding was detected by chemiluminescence (Amersham ECL prime, GE Healthcare) on a Bio-Rad Chemidoc system equipped with ImageLab Software (BioRad). The following antibodies were used: rat anti-CD44 (Abcam, clone IM7), mouse anti-β-actin (Abcam, clone AC-15), and mouse anti-E-selectin (Developmental Studies Hybridoma Bank, clone P2H3) with appropriate secondary antibodies conjugated to HRP (Jackson ImmunoResearch).

#### Confocal microscopy and image analysis

Unless otherwise indicated, imaging was performed using a Quorum spinning disc system mounted on a Zeiss Axiovert 200M microscope, using 63x or 100x oil objectives, a 1.5x magnification lens, and a back-thinned EM-CCD camera (C9100-13, Hamamatsu). Acquisitions were controlled by the Volocity software (Perkin-Elmer), exported and processed with MATLAB (MathWorks) for single-particle tracking, or analyzed and quantified using Volocity or ImageJ (NIH) software.

#### Neutrophil rolling and adhesion experiments

RF24 cells pretreated with control, CD44 or spectrin siRNA were grown in microfluidic chambers of the BioFlux system (Fluxion Biosciences, San Francisco, CA) that had been precoated with 0.05 mg/mL of rat-tail collagen type I in 0.02 M acetic acid. Cells were grown to confluence, washed, and activated with 10 ng/mL of TNF-α for 4 h. Neutrophils were isolated from whole blood as previously described (Riedl et al., 2016) and labeled with 2.5 μM calcein (Life Technologies). TNF-α was washed away and calcein-labeled neutrophils were introduced to the activated RF24 monolayers at a flow rate of 1 dyne/cm^2^. Images and video were obtained over 10 min using a 4X objective on a Nikon Ti epifluorescence microscope using a Qimaging CCD camera and NIS software.

#### Fluorescence recovery after photobleaching (FRAP)

FRAP was performed as previously described (Axelrod et al., 1976) on cells expressing E-selectin-GFP or E-selectin-ΔCT-GFP. A circular region of interest (ROI) on the plasma membrane was photobleached using a 405 nm laser (100% intensity, 0.5 s) and fluorescence recovery was monitored for 1 min, until the intensity plateaued. To ensure that we could distinguish between the apical and basolateral membrane during recordings, ROIs were selected above or below the nucleus. Experiments were performed on a Nikon A1R resonance scanning confocal microscope, using a 63X NA 1.4 objective.

### QUANTIFICATION AND STATISTICAL ANALYSIS

The number of experiments and cells quantified are indicated in the individual Figure Legends. The Mann-Whitney test was used to determine *p-value*s between populations of single-cell measurements. For matched samples, Student’s t tests or two-way ANOVA tests were performed.

## Supplementary Material

Video 1

Video 2

1

## Figures and Tables

**Table:** KEY RESOURCES TABLE

REAGENT or RESOURCE	SOURCE	IDENTIFIER
Antibodies		
anti-β-actin (clone AC-15)	Abcam	Cat. # ab6276
anti-CD44 (rat, clone IM7)	Abcam	Cat. #ab218753
anti-P/E-selectin (CD62P/E)	R&D Systems	Cat. #BBA16
anti-E-selectin (CD62E)	Developmental Studies Hybridoma Bank (DSHB)	Cat. # P2H3
anti-caveolin-1	BD Transduction labs	Cat. #610059
anti-βII-spectrin	Santa Cruz Biotechnology	Cat. #sc-136074
anti-βI-spectrin (clone VD4)	Gift from Dr. M. Stankewich	N/A
anti-moesin	Abcam	Cat. #ab151542
anti-HA-biotin, high affinity (clone 3F10)	Millipore Sigma	Cat. #1258167001
anti-GFP-binding proteins (VHH, nanobody)	Chromotek	Cat. # gt-250
Rat isotype control IgG	eBioscience	Cat. #14-4714-82
Streptavidin-HRP	Cell Signaling Technology	Cat. # 3999
Chemicals, Peptides, and Recombinant Proteins		
Hyaluronan-binding complex, biotinylated	Tammi et al., 1994	N/A
Hyaluronidase from bovine testes Type I-S	Millipore Sigma	Cat. # H3506
Recombinant Tumor Necrosis Factor α	Peprotech	Cat. #300–01A
Phalloidin-Alexa 488	ThermoFisher	Cat. # A12379
Phalloidin-Rhodamine	ThermoFisher	Cat. # R415
Rat tail collagen type I	Millipore Sigma	Cat. # C-7661
Latrunculin A	Sigma Aldrich	Cat. # L5163
Methyl-β-cyclodextrin (MPCD)	Sigma Aldrich	Cat. # 4555
Critical Commercial Assays		
BioFlux	Fluxion	N/A
Experimental Models: Cell Lines		
Human RF24	Fontijn et al., 1995	N/A
Human HUVEC	Lonza	Cat. #C2519A
Human HAMEC	ScienCell	Cat. # 7200
Human BOEC	Isolated as in Noone et al., 2016.	N/A
Mouse RAW 264.7 macrophage	ATCC	ATCC: TIB-71
Oligonucleotides		
CD44 siRNA	ThermoFisher	Cd44HSS101596 Cat No.1299001
Spectrin β-I (SPTB) siRNA	Dharmacon	Cat No. L-017692-00-0005
Spectrin β-II (SPTBN1) siRNA	Dharmacon	Cat No. L-018149-01-0005
Murine CD44wt-GFP	Freeman et al., 2018	N/A
Murine CD44ΔCT-GFP	Freeman et al., 2018	N/A
N-terminal HA-tagged transmembrane actin-binding domain (ezrin)	Freeman et al., 2018	N/A
N-terminal HA-tagged transmembrane actin-binding domain (ezrin) R579A	Freeman et al., 2018	N/A
Caveolin-1-RFP	Hirama et al., 2017	N/A
E-selectin-GFP	This paper	N/A
E-selectin-ΔCT-GFP	This paper	N/A
Software and Algorithms		
Divide and Conquer Moment-Scaling Spectrum	Vega et al., 2018	http://www.utsouthwestern.edu/labs/jaqaman/software
MATLAB	MathWorks	https://www.mathworks.com/products/matlab.html
u-Track	Jaqaman et al., 2008	http://www.utsouthwestern.edu/labs/danuser/software
Volocity	PerkinElmer	https://www.perkinelmer.com
ImageJ	NIH	https://imagej.nih.gov/ij/

## Highlights

- Spectrin confers stability to the apical/luminal endothelial cytoskeleton
- The spectrin network tethers apical CD44, which functions as a “picket”
- The CD44-spectrin “picket fence” restricts mobility of selectins within caveolae
- Disruption of the picket fence abrogates leukocyte rolling and adhesion
