# Supplementary material for: Stabilization of Endothelial Receptor Arrays by a Polarized Spectrin Cytoskeleton Facilitates Rolling and Adhesion of Leukocytes
Source: Cell Rep. Author manuscript; Available in PMC 2020 Oct 11. (PMC7548125; doi:10.1016/j.celrep.2020.107798)
Supplement: 1 [file NIHMS1633236-supplement-1.pdf]

Cell Reports, Volume *31*

Supplemental Information

Stabilization of Endothelial Receptor Arrays by a Polarized Spectrin Cytoskeleton Facilitates Rolling and Adhesion of Leukocytes

Sivakami Mylvaganam, Magdalena Riedl, Anthony Vega, Richard F. Collins, Khuloud Jaqaman, Sergio Grinstein, and Spencer A. Freeman

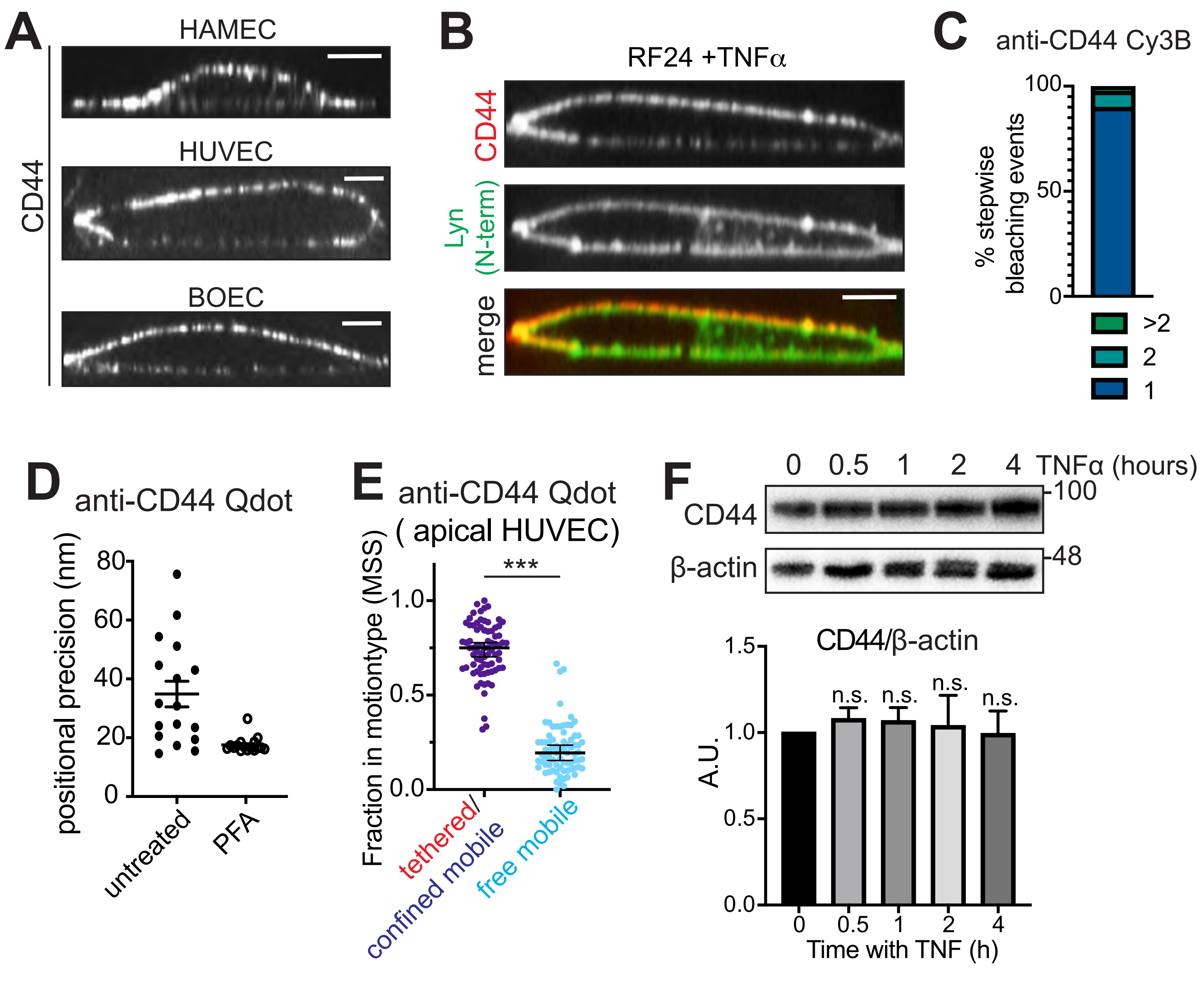

A
HAMEC
HUVEC
BOEC
CD44
B
RF24 +TNFα
CD44
Lyn (N-term)
merge
C
anti-CD44 Cy3B
% stepwise bleaching events
100
50
0
>2
2
1
D
anti-CD44 Qdot
positional precision (nm)
80
60
40
20
0
untreated
PFA
E
anti-CD44 Qdot (apical HUVEC)
Fraction in motiontype (MSS)
1.0
0.5
0.0
***
tethered/ confined mobile
free mobile
F
0 0.5 1 2 4 TNFα (hours)
CD44
β-actin
-100
-48
CD44/β-actin
A.U.
1.5
1.0
0.5
0.0
n.s.
n.s.
n.s.
n.s.
0 0.5 1 2 4
Time with TNF (h)

Supplemental Figure 1 (relates to Figure 1). *Single-particle tracking of CD44.* **A)** Representative orthogonal section of human adipose microvascular endothelial cells (HAMEC; *top*), human umbilical vein endothelial cells (HUVEC; *middle*) and blood outgrowth endothelial cells (BOEC; *bottom*), immunostained for CD44. **B)** Representative orthogonal section of TNFα-treated endothelial cells expressing the N-terminal domain of Lyn-GFP (green) and immunostained for endogenous CD44 (red). **C)**Endothelial cells were incubated with 10 ng/mL of Cy3B-labelled anti-CD44 Fabs. The number of stepwise bleaching events for randomly selected particles that underwent photobleaching on the cell surface while being recorded for 10s at 10 Hz. **D)** The mean positional precision of ≥1000 Qdot labelled particles on untreated or PFA-treated cells, as previously described (Freeman et al., 2018) determined for >1000 events from ≥15 cells. **E)** Modes of apical CD44 mobility on primary HUVEC cells as determined using MSS analysis. Data and means from 3 experiments, each quantifying ≥15 cells**. F)** Endothelial cells treated with 10 ng/mL of TNFα for 0, 0.5, 1, 2 or 4 h were lysed and CD44 expression was assessed by immunoblotting and compared to actin. *Top:* Representative immunoblot. *Bottom:* Quantification of relative CD44 expression by densitometric analysis of 5 independent experiments.

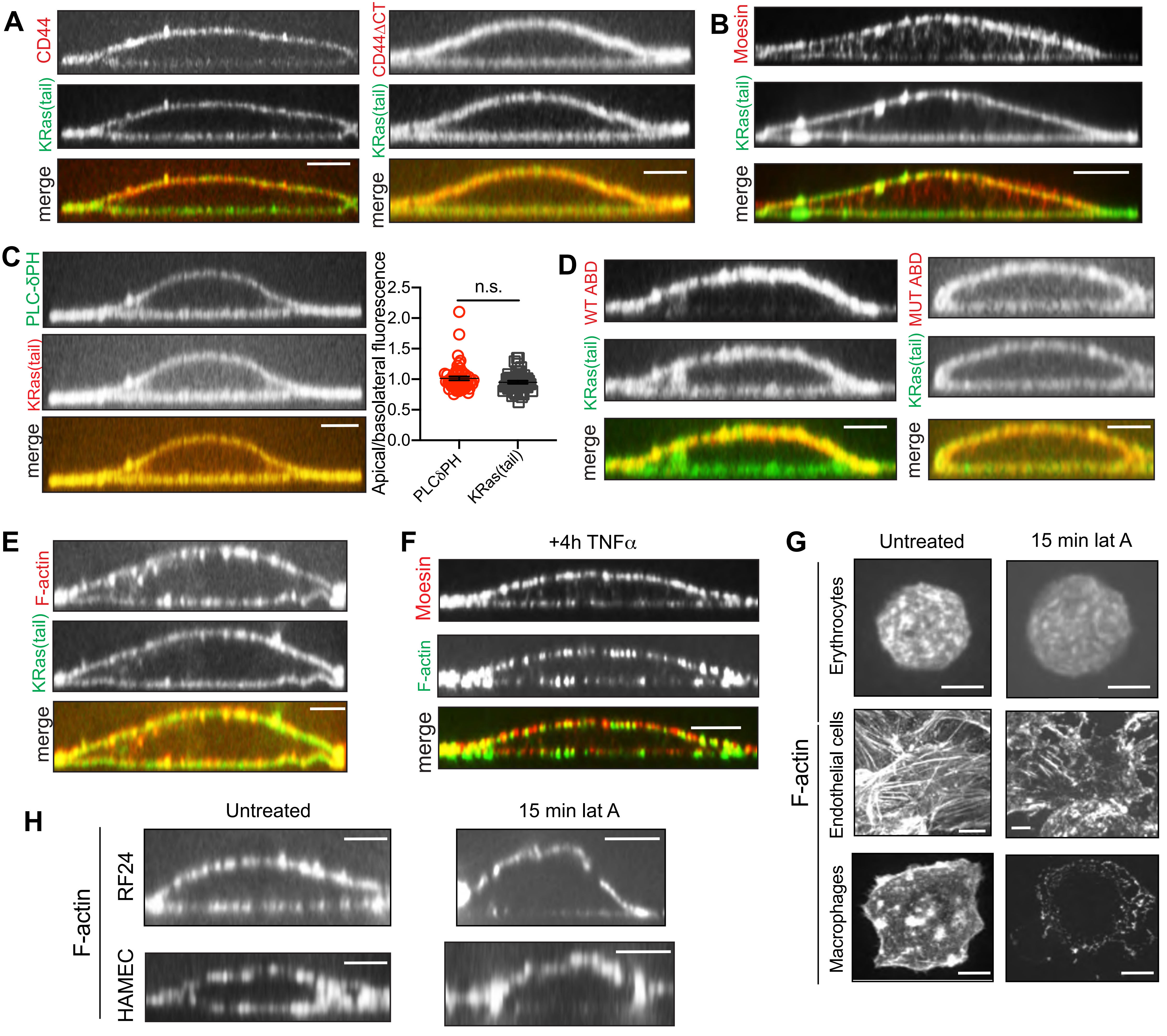
A
CD44
KRas(tail)
merge
CD44ΔCT
KRas(tail)
merge
B
Moesin
KRas(tail)
merge
C
PLC-δPH
KRas(tail)
merge
Apical/basolateral fluorescence
n.s.
2.5
2.0
1.5
1.0
0.5
0.0
PLCδPH
KRas(tail)
D
WT ABD
KRas(tail)
merge
MUT ABD
KRas(tail)
merge
E
F-actin
KRas(tail)
merge
F
+4h TNFα
Moesin
F-actin
merge
G
Untreated
15 min lat A
Erythrocytes
Endothelial cells
Macrophages
F-actin
H
Untreated
15 min lat A
F-actin
RF24
HAMEC

Supplemental Figure 2 (relates to Figure 2). *Unique properties of the apical endothelium.* **a)** Representative orthogonal section of endothelial cells expressing either full-length CD44-mCherry or CD44ΔCT-mCherry (red) and KRas(tail)-GFP (green). **b)** Representative orthogonal section of endothelial cells expressing KRas(tail)-GFP (green) and immunostained for endogenous moesin (red). **c)** *Left*: Representative orthogonal section of endothelial cells expressing the PtdIns(4,5)P_2 probe PLC-δ-PH (red) and KRas(tail)-GFP (green). *Right*: ratio of apical/basolateral membrane fluorescence of both markers for ≥25 cells from 3 experiments. **d)** A transmembrane actin-binding protein that contained the actin-binding domain (ABD) of ezrin fused to the transmembrane domain of Fc receptor, tagged with hemagglutinin for detection was expressed. An otherwise identical construct bearing a R579A mutation in the ABD was used as an inactive control. Representative orthogonal sections of endothelial cells expressing either the chimeric proteins with the wildtype (WT) ABD or mutant (MUT) ABD (red) and KRas(tail)-GFP (green). **e)** Representative orthogonal section of endothelial cells expressing KRas(tail)-GFP (green) and stained with phalloidin (red) to determine F-actin distribution. **f)** Representative orthogonal section of TNFα-activated endothelial cells immunostained for moesin (red) and stained with phalloidin for F-actin (green). G) Representative extended focus confocal images of erythrocytes, endothelial cells and macrophages that were either untreated (left panel) or treated for 15 min with 1 μM LatA. **g)** Orthogonal section of RF24 cell (top) or HAMEC cell (bottom) either untreated or treated for 15 min with 1 μM LatA, followed by fixation, permeabilization and staining with phalloidin.

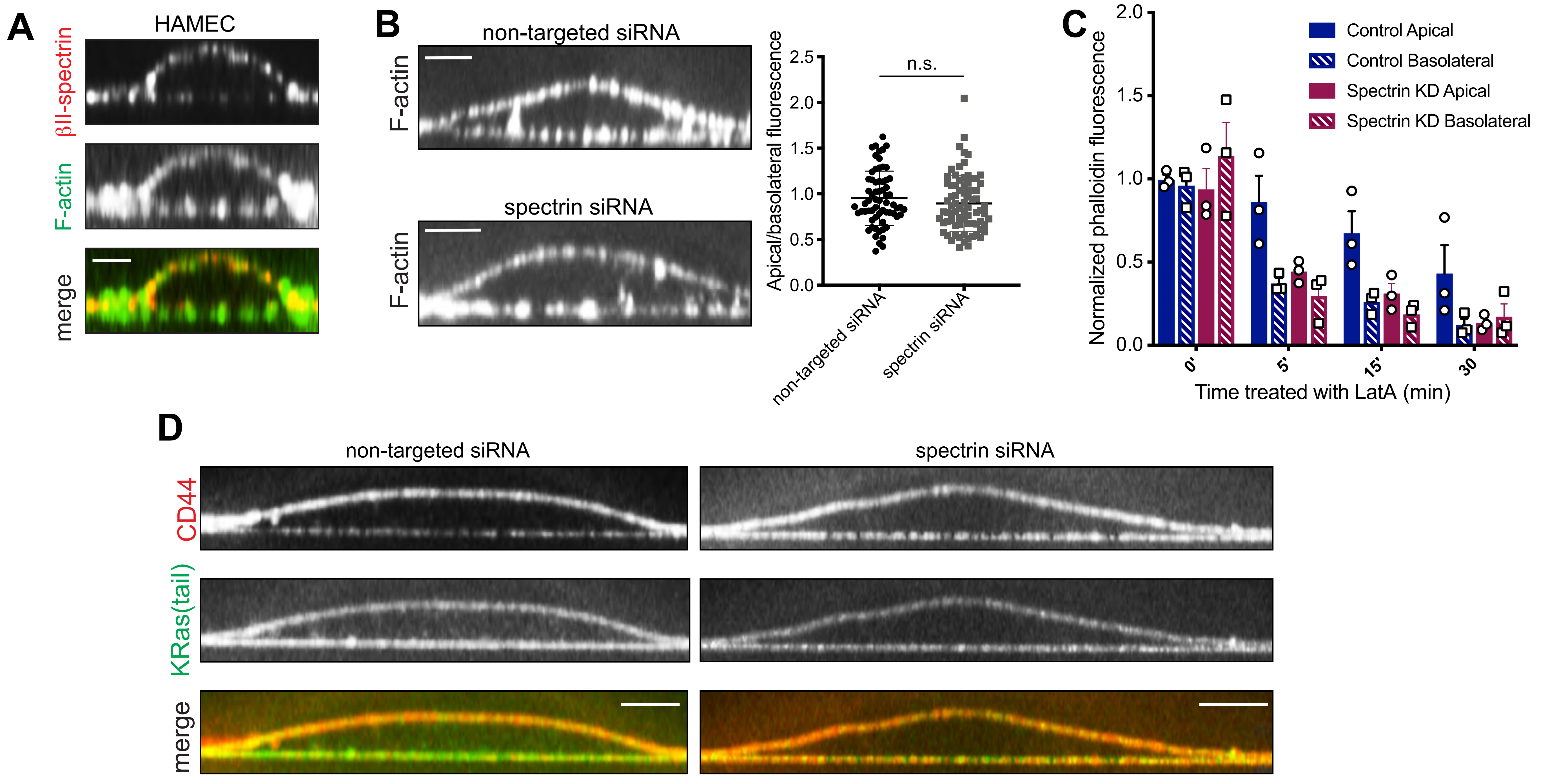
A
HAMEC
βII-spectrin
F-actin
merge
B
non-targeted siRNA
F-actin
spectrin siRNA
F-actin
Apical/basolateral fluorescence
2.5
2.0
1.5
1.0
0.5
0.0
n.s.
non-targeted siRNA
spectrin siRNA
C
Normalized phalloidin fluorescence
2.0
1.5
1.0
0.5
0.0
Control Apical
Control Basolateral
Spectrin KD Apical
Spectrin KD Basolateral
0'
5'
15'
30
Time treated with LatA (min)
D
non-targeted siRNA
spectrin siRNA
CD44
KRas(tail)
merge

Supplemental Figure 3 (relates to Figure 3). *Apical spectrin maintains stability of actin filaments and regulates polarized distribution of CD44. A)* Representative orthogonal section of HAMEC cell immunostained for βII-spectrin (red) and stained with phalloidin for F-actin (green). **b)** *Left:* Representative orthogonal section of endothelial cell treated with control or β-spectrin siRNA and then fixed, permeabilized and stained with phalloidin. *Right*: ratio of apical/basolateral membrane phalloidin fluorescence and KRas(tail)-GFP for ≥20 cells from 3 experiments. **c)** Comparison of phalloidin intensity at the apical and basolateral plasma membranes of endothelial cells treated with control or β-spectrin siRNA following 5, 15 or 30 min of 1 μM LatA treatment, normalized to phalloidin intensity of untreated cells. From ≥30 cells from 3 experiments. **d)** Representative orthogonal sections of endothelial cells treated with control or βI+ βII-spectrin siRNA, expressing KRas(tail)-GFP (green) and immunostained for endogenous CD44 (red).

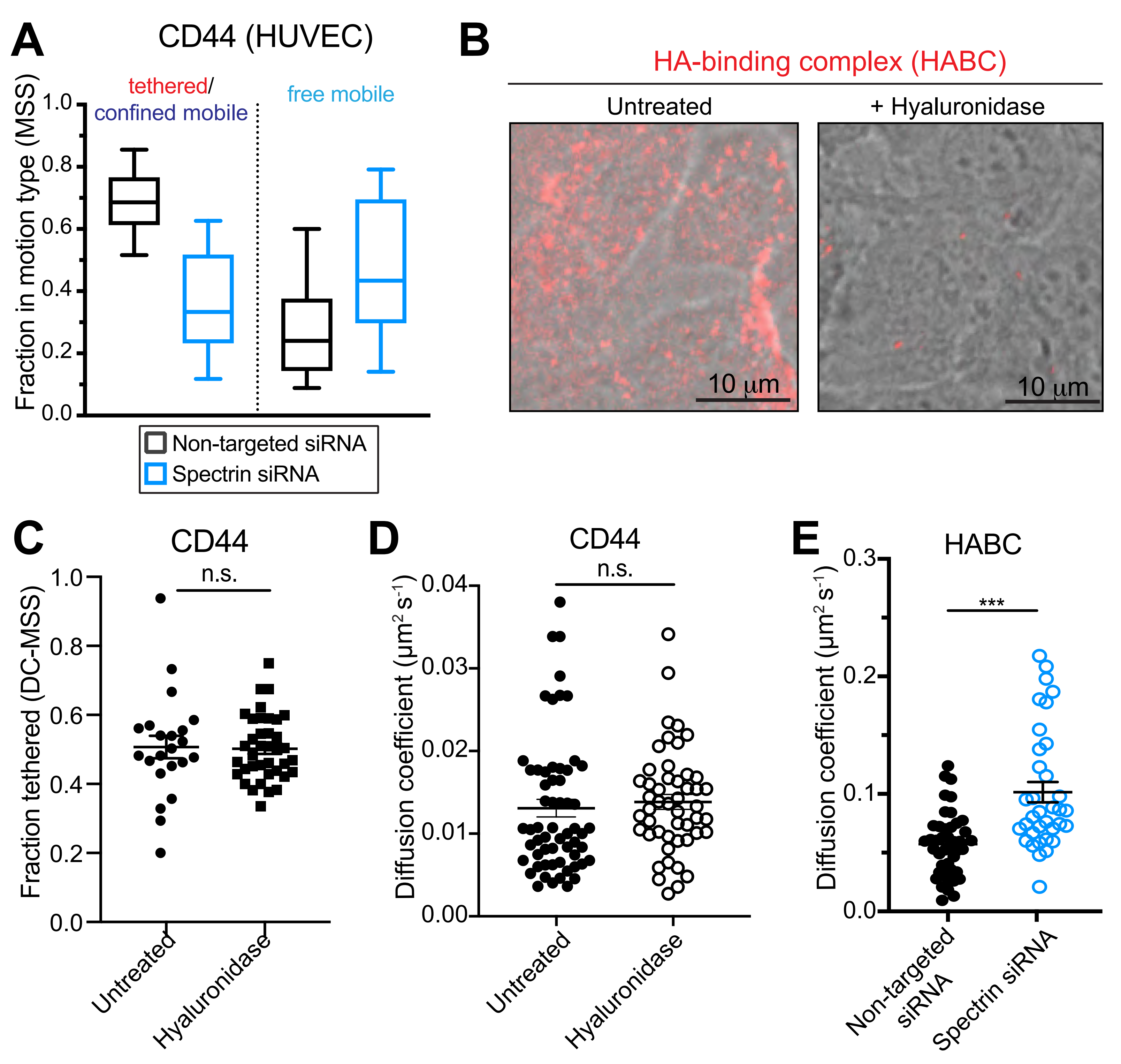
A
CD44 (HUVEC)
tethered/
confined mobile
free mobile
Fraction in motion type (MSS)
1.0
0.8
0.6
0.4
0.2
0.0
Non-targeted siRNA
Spectrin siRNA
B
HA-binding complex (HABC)
Untreated
+ Hyaluronidase
10 μm
10 μm
C
CD44
n.s.
Fraction tethered (DC-MSS)
1.0
0.8
0.6
0.4
0.2
0.0
Untreated
Hyaluronidase
D
CD44
n.s.
Diffusion coefficient (μm² s⁻¹)
0.04
0.03
0.02
0.01
0.00
Untreated
Hyaluronidase
E
HABC
***
Diffusion coefficient (μm² s⁻¹)
0.3
0.2
0.1
0.0
Non-targeted siRNA
Spectrin siRNA

Supplemental Figure 4 (relates to Figure 4). *Spectrins can regulate the stability of HA.* **a)** Modes of apical CD44 mobility on primary HUVEC cells treated with non-targeted or spectrin siRNA, as determined using MSS analysis. Data from 2 experiments, each quantifying ≥20 cells**. b)** Untreated or hyaluronidase-treated RF24 cells were incubated with fluorescent HA-binding complex and imaged. Scale bars: 10 µm. **c and d)** RF24 cells were treated as indicated and incubated with anti-CD44 Fab labeled with Qdots. Qdots labelling the apical surface cells were tracked for 30 s at 33 Hz. Fraction of time tethered (**c**) and diffusion coefficient (**d**) for ≥30 cells. **e)** RF24 cells were treated with control or βI+ βII-spectrin siRNA for 48 h. Cells were then incubated with biotinylated HA-binding protein, labelled with streptavidin-coated Qdots and tracked for 30 s at 33 Hz. Mean diffusion coefficient for ≥20 cells for 3 experiments.

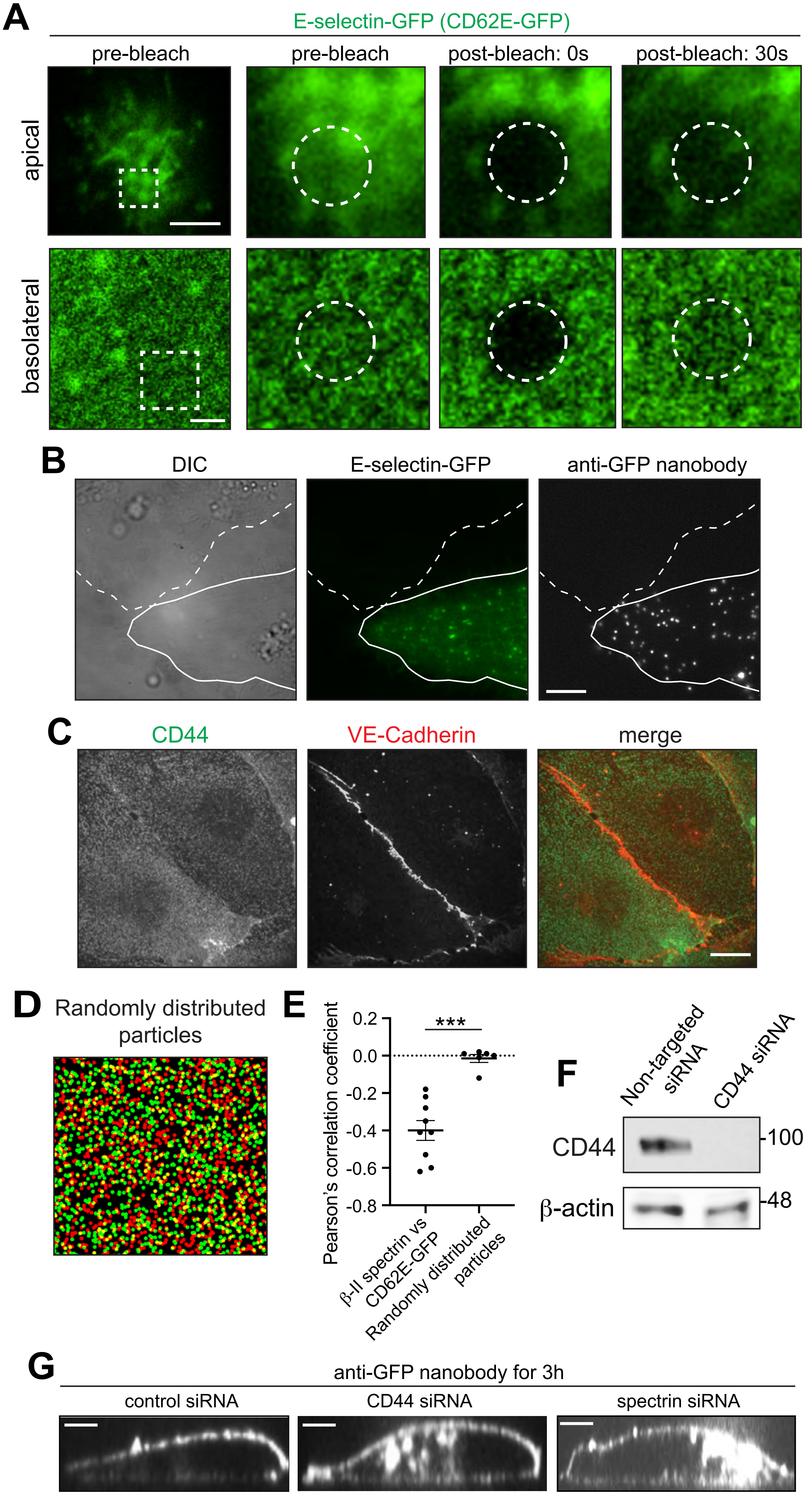
A
E-selectin-GFP (CD62E-GFP)
pre-bleach
pre-bleach
post-bleach: 0s
post-bleach: 30s
apical
basolateral
B
DIC
E-selectin-GFP
anti-GFP nanobody
C
CD44
VE-Cadherin
merge
D
Randomly distributed particles
E
Pearson's correlation coefficient
0.2
0.0
-0.2
-0.4
-0.6
-0.8
***
β-II spectrin vs CD62E-GFP
Randomly distributed particles
F
Non-targeted siRNA
CD44 siRNA
CD44
β-actin
-100
-48
G
anti-GFP nanobody for 3h
control siRNA
CD44 siRNA
spectrin siRNA

Supplemental Figure 5 (relates to Figure 5). *CD44 and spectrin restrict E-selectin diffusion.* **a)** Representative images from FRAP series of E-selectin-GFP (CD62E-GFP) at apical and basolateral membranes. **b)** RF24 cells expressing E-selectin-GFP (CD62E-GFP), incubated with anti-GFP nanobody-Cy3B. **c)** Representative extended projections of confocal images where cells were immunostained for CD44 (green) and VE-cadherin (red). **d)** Representative image of 2000 randomly distributed points. **e)** Pearson's correlation coefficient of individual cells imaged STED, compared to coefficient for images of randomly distributed points. **f)** E-selectin-GFP-expressing cells were treated with control, CD44 or βI+ βII-spectrin siRNA. Representative orthogonal sections following incubation with anti-GFP nanobody for 3 h.

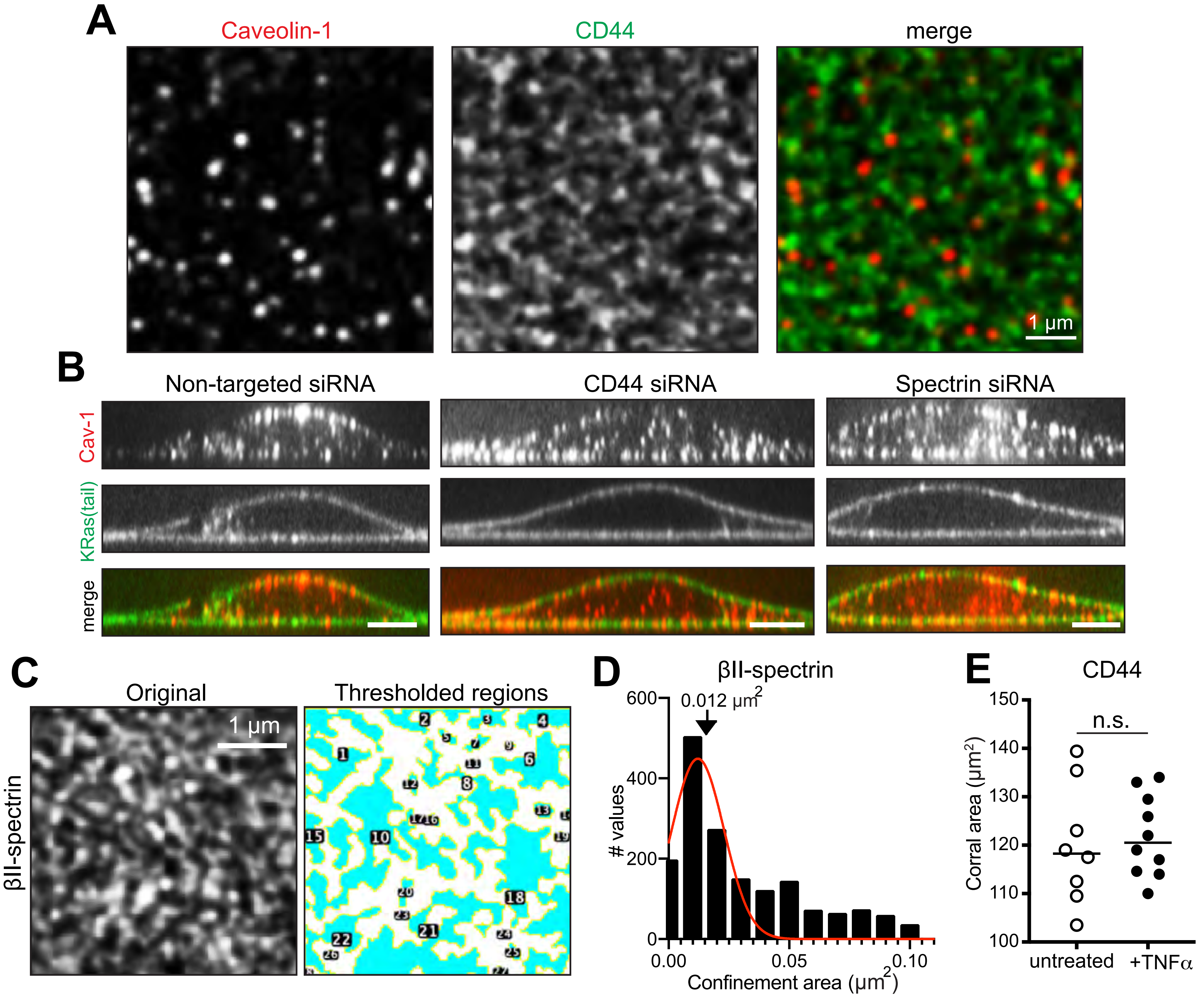
A
Caveolin-1
CD44
merge
1 μm
B
Non-targeted siRNA
CD44 siRNA
Spectrin siRNA
Cav-1
KRas(tail)
merge
C
Original
Thresholded regions
βII-spectrin
1 μm
D
βII-spectrin
0.012 μm^2
values
Confinement area (μm^2)
E
CD44
n.s.
Corral area (μm^2)
untreated
+TNFα

Supplemental Figure 6 (relates to Figure 6). *CD44 and spectrin confine caveolar mobility.* **a)** Endothelial cells stained for CD44 (green) and caveolin-1 (red) and imaged by STED. **b)** Representative orthogonal sections of cells expressing KRas(tail) (green) and stained for CD44 (red) following 48 h treatment with control, CD44 or βI+ βII-spectrin siRNA. **c and d)** Cells were stained for βII-spectrin and imaged using STED. STED images were analyzed using ImageJ software as shown in (**c**). Histogram representing measured confinement areas (**d**) for ≥10 cells. Average confinement area was 0.012 μm^2. **e)** Comparison of CD44 corral area in TNFα-treated or untreated cells.
